# *In silico* evidence of monkeypox F14 as a ligand for the human TLR1/2 dimer

**DOI:** 10.3389/fimmu.2025.1544443

**Published:** 2025-03-17

**Authors:** Ankita Chakraborty, Nabarun Chandra Das, Parth Sarthi Sen Gupta, Saroj Kumar Panda, Malay Kumar Rana, Srinivasa Reddy Bonam, Jagadeesh Bayry, Suprabhat Mukherjee

**Affiliations:** ^1^ Integrative Biochemistry and Immunology Laboratory (IBIL), Department of Animal Science, Kazi Nazrul University, Asansol, West Bengal, India; ^2^ School of Bioscience and Bioengineering, D.Y. Patil International University, Pune, India; ^3^ Department of Chemistry, Indian Institute of Science Education and Research, Berhampur, Odisha, India; ^4^ Vaccine Immunology Laboratory, Department of Applied Biology, CSIR-Indian Institute of Chemical Technology, Hyderabad, Telangana, India; ^5^ Academy of Scientific and Innovative Research, Ghaziabad, India; ^6^ Department of Biological Sciences & Engineering, Indian Institute of Technology Palakkad, Palakkad, Kerala, India

**Keywords:** monkeypox, immunopathogenesis, toll-like receptor, molecular dynamics simulation, *in silico* approach, pathogenesis, emerging virus disease, bioinformatics

## Abstract

Recent emergence of zoonotic monkeypox virus (Mpox) in human has triggered the virologists to develop plausible preventive measures. Hitherto, our understanding on the mechanism of immunopathogenesis of Mpox infection is elusive. However, available experimental evidences suggest induction of inflammation as the main cause of pathogenesis. Toll-like receptors (TLRs) are critical in initiating and modulating the host immune response to pathogens. Inflammatory responses observed in various poxvirus infections have, in fact, been shown to be mediated through TLR activation. Therefore, by in silico approaches, this study seeks to identify the Mpox antigen(s) (MAg) that are most likely to interact with human cell-surface TLRs. The Mpox proteomics data available in UniProt database contain 174 protein sequences, among which 105 immunoreactive proteins were modeled for 3D structure and examined for comparative protein-protein interactions with the TLRs through molecular docking and molecular dynamics simulation. F14, an 8.28 kDa infective protein of Mpox, was found to exhibit strong binding affinity (ΔG=-12.5 Kcal mol^-1^) to TLR1/2 dimer to form a compact thermodynamically stable protein complex. Interestingly, a significant level of conformational change was also observed in both F14 and TLR6 while forming F14-TLR1/2 complex. Based on these data we propose F14 as a putative ligand of human TLR1/2 to initiate proinflammatory signaling in the Mpox-infected host.

## Introduction

Following the dreaded COVID-19 pandemic (2019), a sudden re-emergence of monkeypox virus (Mpox) was reported in 2022 (1). This is also a zoonotic virus, belonging to the Orthopoxvirus genus of the Poxviridae family. It contains a linear double-stranded DNA genome (197.209 bp) and was first isolated in 1958 from the apparent vesiculo-pustular lesions of captive cynomolgus (crab-eating) monkeys in Denmark (2). Twelve years later, human Mpox infection was first recorded in a 9-months old boy in The Democratic Republic of Congo (3–5). Mpox infecting humans is classified into the Central African/Congo Basin (CA) (Clade I) and West African (WA) (Clade II) clades, the latter being the causative agent of 2022 outbreak (6). On August 14^th^ 2024, the World Health Organization (WHO) declared the outbreak as a Public Health Emergency of International Concern (PHEIC) (7). California confirmed its first clade I case in November 2024, and the second was from Georgia on January 14^th^, 2025, both connected with travel histories (8). As per the latest update available in the public domain, outbreaks of clade I virus are still occurring in Central and Eastern Africa, and the ongoing clade II (subclade b) outbreak has claimed more than 100,000 victims from 122 total countries, of which 115 countries had no previously reported cases of Mpox (8, 9).

The incidence of Mpox between 2022-2024 displayed varying degrees of impact. Prior to 2022, the disease was largely neglected, with a slight decline in cases observed in 2023. However, since April 2024, there has been a resurgence in cases and a shift in the epidemiology, with 27 countries reporting new cases (10). The WHO’s External Situation Report, released on June 10, 2023, confirmed that all six WHO regions were affected by Mpox from January 2022 to June 2023 (11). While clade IIb has been identified as the primary strain responsible for the ongoing outbreak, clade I Mpox has increasingly affected all age groups, particularly infants and children (10). Since July 2024, mutated clades Ia, Ib and IIb are being considered to cause the series of outbreaks within and outside Africa (11). These variant strains are associated with alarming changes in epidemiological patterns, clinical symptoms, and higher mortality rates.

Notably, the Democratic Republic of Congo (DRC) has seen a significant shift toward human-to-human transmission, with new cases reported in provinces that had previously not seen Mpox infections (10). As of May 2024, 23 provinces in the DRC had at least one suspected case, with 7,000 clinically compatible cases and a fatality rate of 5.3% reported for that year (10). Children and adolescents under the age of 15 accounted for 67% of these cases and 84% of the fatalities. Neonates, particularly those under one year old, are considered to be four times more likely to die from the infection than those over 15 years old (10).

By January 1, 2024, Mpox cases had been reported in 73 countries, including Sweden, Belgium, Italy, France, Germany, the Netherlands, Israel, Switzerland, Spain, Portugal, Denmark, the Czech Republic, and Canada (11). Between February and July 2024, African regions were the most affected, with 3,061 cases and 23 deaths. However, by September 2024, America had reported the highest number of cases (65,877), followed by Europe (28,176), Africa (9,425), the Western Pacific (4,379), Southeast Asia (971), and the Eastern Mediterranean region (871) (12, 13). In October 2024 alone, Africa reported approximately 10,944 laboratory-confirmed cases (14). This fluctuating epidemiological trend throughout 2024 poses significant challenges to the WHO’s proposal titled “Strategic Framework for Enhancing Prevention and Control of Mpox 2024–2027,” which aims to curb and ultimately eliminate Mpox by 2024-2025 (15, 16).

The signs and symptoms of Mpox infection resemble those of smallpox and varicella infections, including fever, headache, myalgia, external or internal rashes lasting for about a fortnight up to a month. Lymphadenopathy, particularly in the maxillary, cervical or inguinal (most prominent) lymph nodes is also common and can last 2-4 weeks (17–20). However, the mortality rate for Mpox is lower (3.6 to 10.6%, particularly in endemic regions) compared to smallpox (21). Mpox-infected individuals are also prone to secondary infections like encephalitis, gastrointestinal and skin infections, respiratory complications, bronchopneumonia, sepsis and corneal infections, which can lead to blindness (18, 22). Recently, proctitis (painful sores and swelling in the rectum) and difficulty in micturition have been noted as additional symptoms (23). While Mpox is typically a self-limiting illness, it can be more severe in children, pregnant women, and immunocompromised individuals, such as those with HIV. The virus is transmitted through respiratory droplets, bodily fluids, close contact with lesions, or contaminated items used by infected individuals (24, 25).

Mpox pathogenesis is believed to follow a mechanism similar to that of other Orthopoxviruses (e.g., Vaccinia virus), utilizing chondroitin sulfate or heparan sulfate-containing host membrane proteins as entry receptors (26–29). Upon invasion, the virus replicates to produce two types of virions: the intracellular mature virion (IMV), which mediates inter-host transmission, and the extracellular enveloped virion (EEV), which has a fragile outer membrane and facilitates inter-cell transmission within the host (25). In human-to-human transmission, the respiratory and oropharyngeal mucosa serve as the primary inoculation sites and after an incubation period of 7-14 days, virus replication starts (25). The viral load spreads to nearby lymph nodes, causing primary viremia. This phase is followed by the highly infectious prodromal stage, leading to secondary viremia, which results in viral invasion of distal lymph nodes, the skin, and tertiary lymphoid organs. During this phase, mucocutaneous lesions, lymphadenopathy, and other nonspecific symptoms typically appear. A re-emergent episode may present with a prodromal phase that features mild or almost unnoticeable symptoms before the onset of lesions (30).

The characteristic fever lasts up to 3 days, followed by the appearance of painful rashes. These rashes typically begin on the face and spread across the body in a centrifugal pattern, starting with enanthem (lesions on the tongue and mouth), progressing to macular (flat patches beginning on the face and spreading to the limbs), papular (raised lesions), vesicular (papules filled with clear fluid), and pustular (sharp, round, firm vesicles filled with opaque fluid) stages, before eventually crusting up. This is followed by a pruritic desquamation phase (18). Dermal histopathology reveals progressive ulceration and necrosis, along with destruction of sebaceous glands and hair follicles, indicating significant inflammatory involvement (25).

Considering the inflammatory pathology of Mpox infection, an obvious question is the identity of possible viral antigen and its possible interacting receptor in the human immune system that mediate host-virus interaction to induce the initial inflammatory signal. In this context, human cell-surface toll-like receptors (TLRs) play a key role in recognizing the viral antigens and inducing the expression of the inflammatory cytokines, chemokines and interferons (31–34). TLRs have been shown to play a major role in the pathogenesis of various emerging viral diseases like COVID-19 (35, 36). Inflammatory responses in different poxvirus infections have been found to be TLR-mediated (37). Extensive genomics and proteomics studies on Mpox have revealed the presence of several virulent genes/proteins that have significant immunomodulatory functions (38). However, the mechanistic insights on the Mpox pathogenesis are still illusive. Therefore, by *in silico* approaches, we aimed to identify the Mpox antigen(s) (MAgs) that most likely mediate the host virus-interactions by acting as a ligand(s) for the human cell-surface TLRs.

## Methods

### Data mining

The proteome (accession ID: UP000516359) of the Mpox was retrieved from the UniProt database (https://www.uniprot.org/), and antigenic proteins of Mpox (IMV and/or EEV subtypes) were selected and extracted in FASTA format for further analyses (**Supplementary Table S1**). The crystal structures of human TLR4-MD2 (PDB ID: 3FXI_AC), TLR1/2 dimer (PDB ID: 2Z7X_AB), and TLR5 (PDB ID: 3J0A) were obtained from protein data bank (https://www.rcsb.org/search) while TLR2/6 dimer was constructed through molecular docking of TLR2 (PDB ID: 3J79_AB) and TLR6 (obtained through homology modeling).

### Homology modeling and assessment of stereochemical quality

3D structure of each of the MAg was modeled from its amino acid sequence employing trRosetta server (https://yanglab.nankai.edu.cn/trRosetta/) (39) and the developed structures were verified for the stability and stereochemical quality by ERRAT, Verify3D, Prove, PROCHECK and WHATCHECK by accessing SAVES 6.0 (https://saves.mbi.ucla.edu/) and ProSA (https://prosa.services.came.sbg.ac.at/prosa.php).

### Molecular docking and analysis of biophysical interactions

To examine the binding of Mpox proteins to human cell-surface TLRs (TLR1/2, TLR2/6, TLR4 and TLR5), molecular docking experiments were conducted employing Hex 8.0.0 software (http://hex.loria.fr/). Based on the docking score (≥ -1000), docked structures comprising MAgs and TLRs were screened, binding energy and affinity were calculated and finally visualized on PyMOL platform (https://pymol.org/2/), while biophysical parameters of protein-protein interactions were analyzed using Discovery Studio (https://discover.3ds.com/).

### Assessment of stability of MAg-TLR complexes

#### Normal mode analysis

MAg-TLR complexes were analyzed for the domain flexibility and stability by NMA, using iMODS (http://imods.chaconlab.org). Stability factors contributing to protein-protein interactions like direction of motion, changes in bond length and angles, perturbation in atomistic fluctuations and others were inferred by studying the NMA in dihedral co-ordinates, following Das et al. (40, 41).

#### Molecular dynamics simulation (MDS)

In addition to NMA, complexes formed by F14 with TLR1/2, F14 with TLR2/6, MPXVgp154 with TLRL1/2, Cowpox A-type inclusion protein with TLR2/6, B11R with TLR2/6 and A47R with TLR2/6 were analyzed for their biophysical and conformational stability at residual and atomic levels within a simulation phase of 100 ns using GROMACS v5.1 (GROningen MAchine for Chemical Simulations) molecular dynamics freeware (https://www.gromacs.org/) following Padma et al. (42). In brief, docked complexes were prepared in an aqueous solvated system followed by system energy minimization and isothermal-isochoric equilibration for 50,000 steps for a final run through a simulation phase of 100 ns following Das et al. (41). Structural stability, compaction and residual flexibility of each protein complex in terms of root-mean-square-deviation (RMSD), root-mean-square-fluctuation (RMSF), radius of gyration (R_g)_, and solvent-accessible-surface-area (SASA) were computed accordingly.

### Calculations of binding free energy

Binding affinity of each MAg to form stable complex with TLR was determined through calculating the binding free energy (ΔG_bind_) of an antigen-TLR complex using GMXPBSA 2.1 suite fitted in GROMACS package following Gorai et al. (43).

### Analysis of conformational change

Occurrence of conformational change as an outcome of protein-protein interactions between the MAgs and interacting TLRs before and after forming TLR-MAg binary complex were determined through superimposition of the 3D structure of the unbound TLRs or MAgs with their respective complexes using PyMOL (https://pymol.org/2/) (40).

### Determining key residues of F14 mediating interactions with TLR1/2

To affirm the significance of the amino acids of F14 in forming F14-TLR1/2, a total of 7 amino acids (GLU34, ASP59, ASP62, ASP63, GLU66, GLU70, and ILE73) were selected to induce random mutations using the Sequence Manipulation Suite (SMS) server (https://www.bioinformatics.org/sms2/) (40). Mutant protein sequences were modeled and docked with TLR1/2 afresh, using trRosetta server and Hex 8.0.0 software respectively.

### In-silico cloning of F14

The cDNA sequence was deduced from its amino acid sequence F14, tagged with 6 His codons, and cloned in pET30ax for recombinant production in *Escherichia coli* following our earlier reports (41, 42).

## Results

### Screening, physicochemical characterization and structure prediction of TLR-interacting MAgs

The mediators of inflammation in Mpox infection remain unclear. Therefore, this study was undertaken to identify the key factors driving Mpox-human cell interactions. TLRs are the innate immune sensors that recognize viral antigens to shape innate as well as adaptive immune responses (44, 45). Considering this, UniProt database was explored to retrieve the human Mpox proteome (accession ID: UP000516359) that resembled a total of 174 protein/peptide sequences. Protein structures of 105 sequences were modeled, excluding the partial sequences. These were evaluated for their potential to serve as Pathogen-Associated Molecular Patterns (PAMPs) for human cell-surface TLRs, specifically TLR1/2, TLR2/6, TLR4, and TLR5, by assessing their binding potential to these receptors (**Supplementary Table S1**). Molecular docking data revealed 79 antigens of 105 to exhibit varying degrees of interaction with cell-surface TLRs (**Supplementary Table S2**). Based on the docking scores that indicate the binding strength of viral proteins to TLRs, initially 10 protein complexes were selected. These were further examined for binding affinity, resulting in a final repertoire of six MAg-TLR complexes: F14-TLR1/2, F14-TLR2/6, MPXVgp154-TLR1/2, cowpox A-type inclusion protein-TLR2/6, B11R-TLR2/6, and A47R-TLR2/6 (**Supplementary Table S2**). Thus, five viral antigens were essentially screened for further studies (**Supplementary Figures S1A, B**).

As depicted in **Supplementary Figure S1C** (i–v), MAgs F14, MPXVgp154, A47R, cowpox A-type inclusion protein and B11R exhibited 91.2%, 88.6%, 90.7%, 90.2% and 90.9% of residues in the most favored regions of their Ramachandran plots, with a negligible percentage of residues in additionally allowed regions and disallowed regions. Their overall quality factors as evaluated by the ERRAT plots (**Supplementary Figure S1D**) were 100.00%, 98.182%, 98.253%, 91.540% and 95.253% respectively. All these data indicate that five viral antigens are stable (**Supplementary Table S3**). Analyses using Verify3D, along with Z-score graphs, further supported these inferences (**Supplementary Figures S2**, **S3**).

### Comparative protein-protein interactions between MAgs and human TLRs

**Figure 1:**
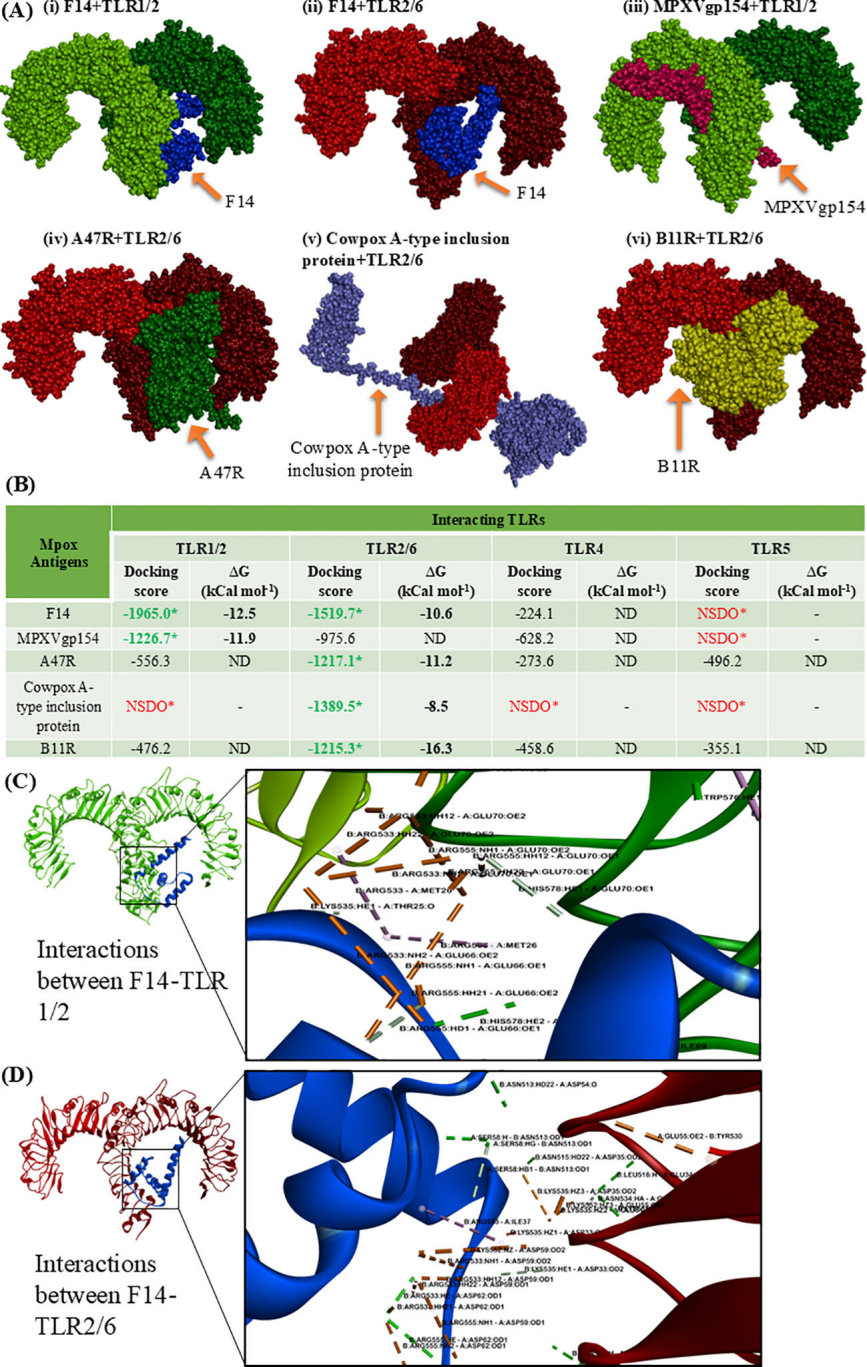
Molecular docking showing the protein-protein interactions between the Mpox antigens (MAgs) and human cell-surface TLRs. **(A)** 3D space-fill models showing the binding topology of six MAgs-TLR complexes, namely (i) F14 + TLR1/2, (ii) F14 + TLR2/6, (iii) MPXVgp154 + TLR1/2 (iv) A47R + TLR2/6, (v) cowpox A-type inclusion protein + TLR2/6 and (vi) B11R + TLR2/6. **(B)** Quantitative data on docking score and binding free energy **(Δ)** involved in forming the MAgs-TLR complexes. *NSDO refers no significant docking observed; ND, not determined. **(C)** Biomolecular interactions occurring in the protein complex formed through the binding of Mpox F14 with human TLR1/2. **(D)** Biophysical interactions occurring within the F14-TLR2/6 complex. Formation of non-covalent interactions like hydrogen bonds, hydrophobic interactions, electrostatic interactions and salt bridges, and other interactions are respectively depicted by green, pink, light orange and gray color.

Following the screening of MAgs for their binding potential to human cell-surface TLRs, comparative analyses of protein-protein interactions between the antigens and TLRs were performed (**Figures 1A, B**). Molecular docking analysis revealed distinct binding patterns of the five MAgs (F14, MPXVgp154, A47R, cowpox A-type inclusion protein and B11R) to the TLRs (**Figure 1A**). Among the five antigens, F14 was found to exhibit a strong binding potential to two distinct cell-surface TLRs, viz. TLR1/2 and TLR2/6 as evidenced by the respective docking scores of -1965.0 and -1519.7, along with the binding energy (-ΔG) of 12.5 and 10.6 kCal mol^-1^ (**Figure 1B**). Although, the other MAgs A47R and B11R showed interactions with all the TLRs of interest, the strength of binding was below the screening limit of -1000 in most of the cases (**Figure 1B**). Owing to its explicit intense binding ability to the human TLR1/2 and TLR2/6, F14 was selected for further studies.

### Mpox F14 as a putative ligand of TLR 1/2 dimer

Preliminary analysis of the binding topology indicated that F14 binds to the extracellular domain of TLR1/2 and TLR2/6 (**Figures 1A, B**), which is a characteristic feature of TLR-ligand. This has prompted us to further investigate the potential of F14 as a putative ligand for the identified TLRs. Inspired by the previous studies in this area (40, 46, 47), we aimed to determine the strength and mode of interactions in F14-TLR complex in comparison with the other MAg-TLR complexes.

### Mpox F14 possesses strong binding affinity to human TLR1/2

**Figure 2:**
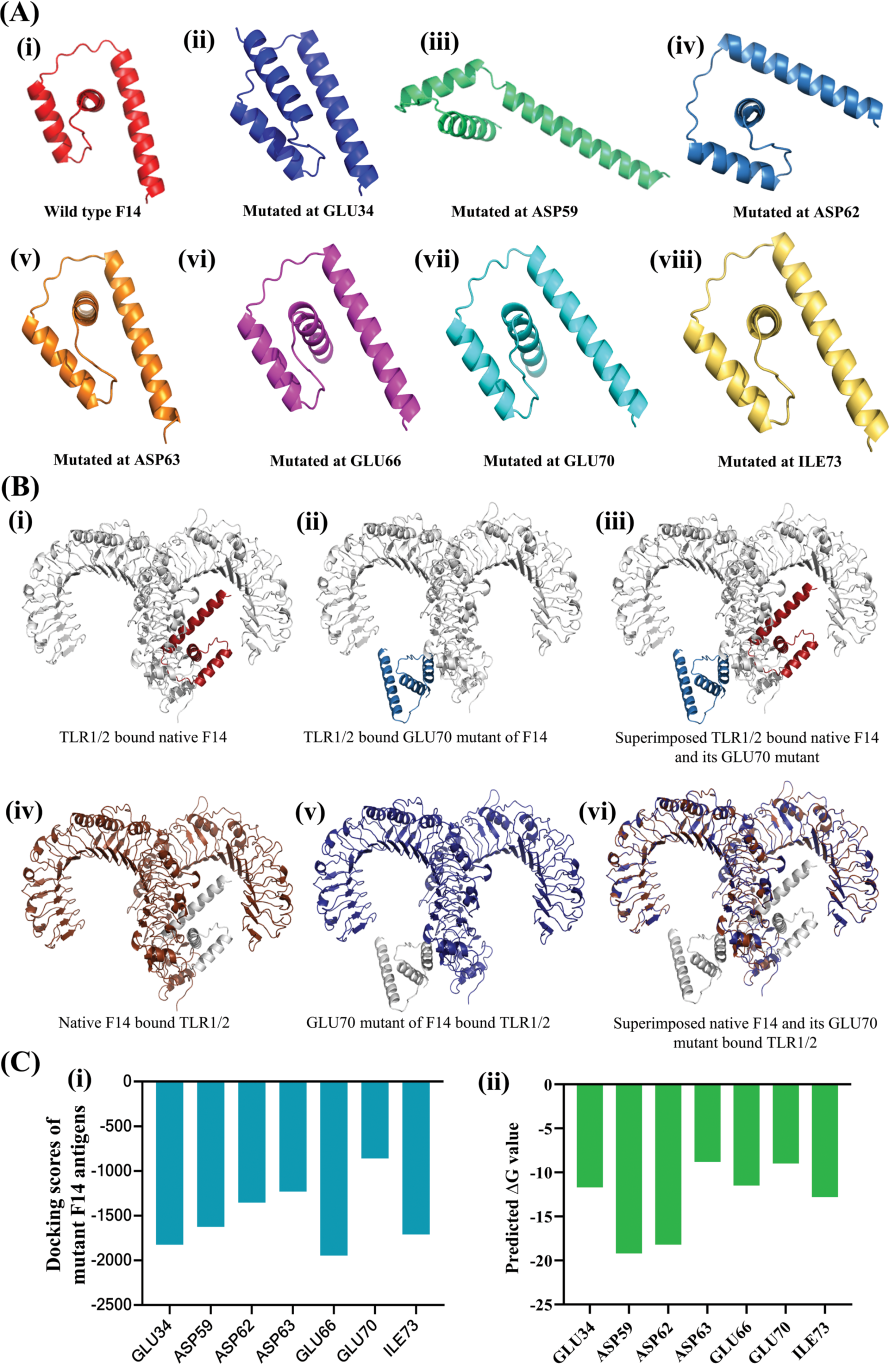
Determining the relative importance of the interacting amino acids in F14 for mediating complex formation with human TLR1/2. **(A)** Variants of F14 generated by induced mutations at specific residue positions, highlighting the shortest bond lengths with corresponding TLR residues. Shown are (i) wild type F14, (ii) mutated at GLU34, (iii) mutated at ASP59, (iv) mutated at ASP62, (v) mutated at ASP63, (vi) mutated at GLU66 (vii) mutated at GLU70 and (viii) mutated at ILE73. **(B)** Effect of in-silico induced random mutations on F14 protein conformation before and after formation of the F14-TLR1/2 complex, comparing wildtype and mutant F14. Changes in protein conformation for wild type F14 and its GLU70 mutant are shown as (i) TLR1/2 bound native F14, (ii) TLR1/2 bound GLU70 mutant of F14 and (iii) superimposed TLR1/2 bound native F14 and its GLU70 mutant. Conformational changes in human TLR1/2 due to binding with native and GLU70-mutant F14 protein are shown as: (iv) native F14 bound TLR1/2, (v) GLU70 mutant of F14-bound TLR1/2 and (vi) Superimposed native F14 and its GLU70 mutant bound to TLR1/2. **(C)** Quantitative data on the protein-protein interactions in the different complexes formed by the binding of various mutants of F14 with TLR1/2: (i) comparative docking scores, and (ii) binding free energy in the formation of F14-TLR complexes.

To gain molecular insights on the strong protein-protein interaction between F14 and TLR1/2 as well as F14 and TLR2/6, we investigated the biophysical interactions occurring in the F14-TLR interface (**Table 1**). Interestingly, the major immunoreactive protein identified in this *in-silico* study, F14, was found to exhibit affinity for both TLR1/2 and TLR2/6 (**Figures 1A, B**; **Table 1**; **Supplementary Table S2**). Therefore, comparative studies were conducted to assess the binding strength and stability of F14 with both TLRs. Molecular docking followed by analyses of binding energy and involvement of different non-covalent forces of attraction collectively revealed that the F14-TLR1/2 interaction is the strongest among the six screened viral protein-TLR complexes (**Figure 1B**; **Table 1**; **Supplementary Table S2**). We identified 31 hydrogen bonds, 6 hydrophobic interactions (all pi-alkyl type) in F14-TLR1/2 complex, while F14-TLR2/6 complex exhibited 26 hydrogen bonds and 4 hydrophobic interactions (2 pi-alkyl type) (**Figures 1C, D**; **Supplementary Table S2**). The biophysical interactions displayed within other MAg-TLR complexes are tabulated in **Supplementary Tables S4** and **S5**. The higher number of hydrogen bonds and hydrophobic interactions at the F14-TLR1/2 interface indicates that this structure is a more strongly held protein complex (**Figures 1C, D**; **Table 1**). To examine the critical role of each amino acid at the F14-TLR1/2 interface in mediating the protein-protein interaction, we generated random mutants of F14 (**Figure 2**). Among the 7 mutants (GLU34, ASP59, ASP62, ASP63, GLU66, GLU70, and ILE73), a significant reduction in F14-TLR1/2 interaction, both in binding topology and binding energy, was observed with the GLU70^-^F14 mutant thus indicating that GLU70 is a critical mediator implicated in the binding of F14 to TLR1/2 (**Figure 2**; **Supplementary Table S6**).

**Table 1:** Biomolecular interactions^*^ between F14-TLR1/2 and F14-TLR2/6.

F14-TLR1/2 complex			F14-TLR2/6 complex		
Residue of F14 protein	Residue of TLR	Distance (Å)	Residue of F14 protein	Residue of TLR	Distance (Å)
Hydrogen bonds					
MET1	GLU422	2.22	LYS2	ASP278	2.55
LYS2	GLU416	2.20	LYS2	ARG304	1.80
TYR6	ASP1011	2.69	SER58	ASN513	2.76
ASN56	ASP1035	2.48	SER58	ASN513	1.59
GLU46	LYS84	2.59	ASP54	ASN489	2.08
ILE73	TRP576	1.73	PHE53	SER492	2.00
GLU66	HIS578	2.23	ASP54	ASN513	2.36
ASP72	GLY605	2.78	ASP35	ASN515	1.98
ASN17	LYS1056	2.13	GLU34	LEU516	2.69
LEU16	LYS1056	2.62	ASP62	ARG533	2.30
ASN56	SER1057	2.05	GLU34	LYS535	2.70
GLU55	ILE1058	2.14	ASP62	ARG555	2.92
SER24	GLN1077	2.56	GLN23	ARG55521	2.83
SER58	LYS1079	2.76	GLN23	ARG55522	2.94
SER58	LYS1079	2.32	GLU31	GLN557	2.32
GLU34	SER1105	1.85	ASP29	THR579	2.76
GLU46	LYS84	2.42	ASP29	LYS608	2.52
THR25	LYS535	2.43	MET26	LYS608	2.92
GLU66	ARG555	2.93	MET26	LYS608	3.03
GLU70	HIS578	2.53	LYS2	ASP278	2.43
ASP72	GLY605	2.76	LYS2	ARG304	2.82
ASP72	GLY605	2.58	SER58	ASN513	2.79
ASN17	LYS1054	2.65	ASP52	ARG442	2.59
ASN17	LYS1054	2.54	PHE53	SER492	2.83
ASP63	LYS1056	3.09	GLU34	ASN534	2.26
GLU55	SER1057	3.04	ASP33	LYS535	3.03
ASN56	SER1057	2.70			
GLU55	SER1057	2.38			
GLU55	VAL1081	2.59			
GLU55	PRO1082	2.52			
ASP29	GLY1131	2.32			
Hydrophobic interactions					
MET26	ARG533	4.69(Pi-Alkyl)	LYS2	ARG304	4.63(Alkyl)
MET26	ARG555	4.74(Pi-Alkyl)	ILE37	ARG533	5.32 (Alkyl)
TYR6	LYS1013	5.00 (Pi-Alkyl)	HIS3	LEU179	5.42 (Pi-alkyl)
LYS2	TYR156	3.96 (Pi-Alkyl)	PHE53	LYS469	4.38 (Pi-alkyl)
ILE73	TRP576	4.20 (Pi-Alkyl)			
ILE69	HIS578	4.36 (Pi-Alkyl)			
Electrostatic interactions					
MET1	GLU227	4.74	LYS2	ASP278	4.31
GLU66	ARG533	4.89	ASP59	ARG533	5.19
GLU70	ARG533	3.87	ASP59	LYS552	4.19
GLU66	ARG555	5.08	ASP59	ARG555	4.50
GLU70	ARG555	4.84	ASP62	ARG555	3.27
GLU34	ARG1108	5.00	ASP27	LYS608	4.41
			PHE53	LYS469	3.98 (Pi-cation)
			GLU55	TYR530	3.88 (Pi-cation)
			HIS3	GLU227	4.33 (Pi-anion)
Salt bridges					
GLU70	ARG533	1.98	MET1	ASP278	1.92
GLU70	ARG533	2.84	LYS2	GLU275	2.49
GLU70	LYS552	1.94	LYS2	GLU275	2.27
GLU70	ARG555	2.28	ASP52	ARG44211	2.09
GLU66	ARG555	1.88	ASP59	ARG53312	2.68
GLU70	ARG555	1.79	ASP62	ARG53321	1.74
ASP63	LYS1056	1.98	ASP59	ARG53322	2.18
ASP59	LYS1079	1.98	ASP33	LYS535	1.69
ASP62	LYS1079	1.76	GLU34	LYS535	2.17
GLU34	ARG1108	1.97	ASP35	LYS535	3.20
GLU34	ARG1108	3.00	GLU55	LYS552	1.83
Others					
MET1	TYR156	5.12			

^*^ To gain molecular insights on the strong protein-protein interaction between F14 and TLR1/2 as well as F14 and TLR2/6, we investigated the biophysical interactions occurring in the F14-TLR interface.

### Mpox F14 forms stable complex with TLR1/2

**Figure 3:**
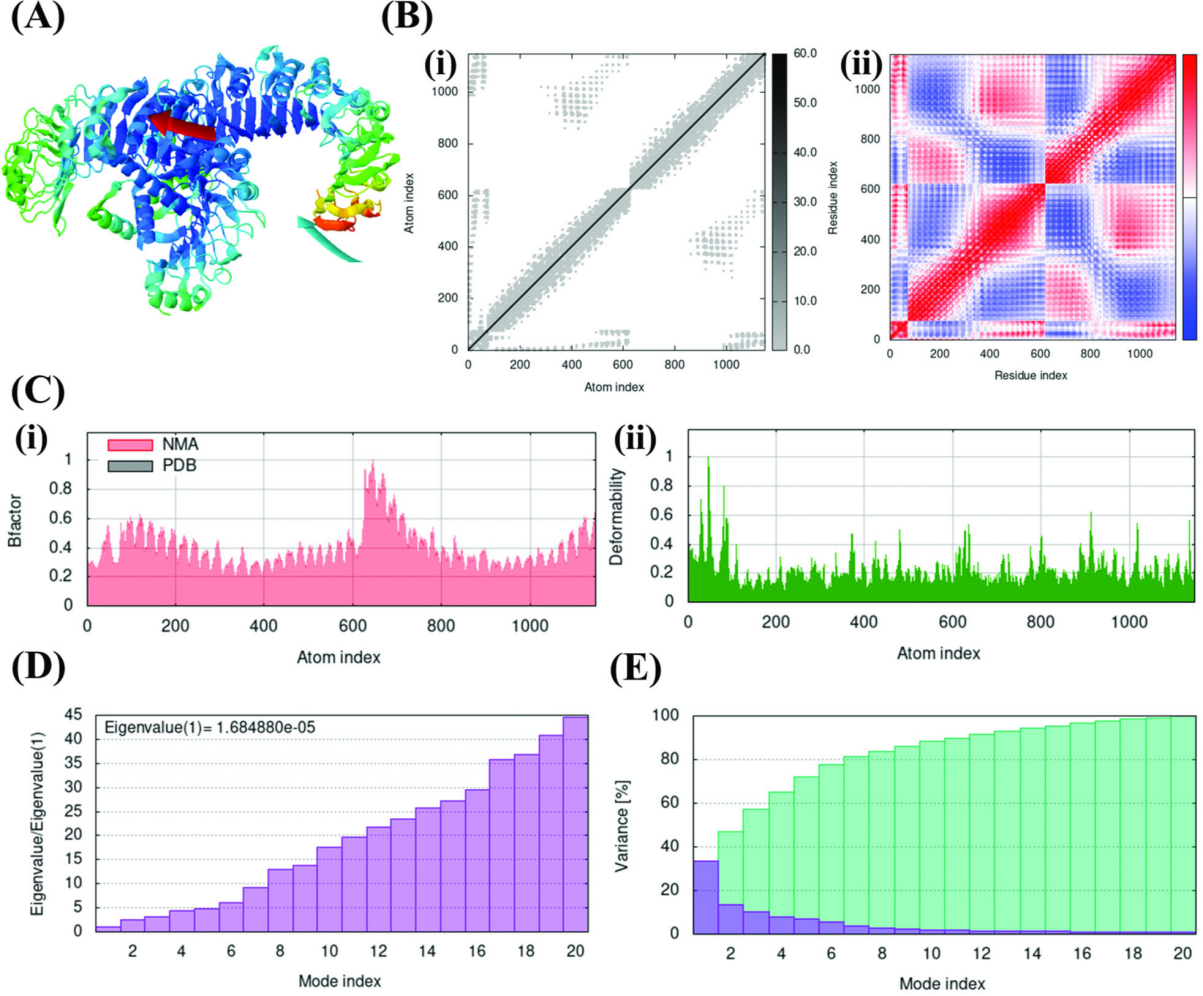
Normal Mode Analysis (NMA) simulation delineating the stability of F14-TLR1/2 complex in terms of domain mobility and flexibility. **(A)** Affine arrow indicating the direction of molecular motion in the F14-TLR1/2 complex. **(B)** Deformation, atomistic and residual fluctuations in the F14-TLR1/2 complex, shown by (i) Elastic network map and (ii) Covariance matrix. **(C)** (i) B-factor graph and (ii) Deformability plot. **(D)** Eigenvalue graph. **(E)** Variance graph where the blue color represents individual variance and the cyan color represents cumulative variance.

After confirming the strong binding affinity of F14 to TLR1/2, the stability of F14-TLR1/2 complex was analyzed at biophysical and thermodynamic levels. First, the domain mobility and flexibility of the two interacting proteins in the MAg-TLR complex was analyzed by NMA (**Figure 3**). By simulating the functional motions within an equilibrium position, this simulation approach provides an insight on the number of flexible conformations of a protein ligand or its putative receptor while occurring as a protein complex (40, 48). Here, the direction of motion of the interacting proteins contributing to domain dynamics is represented by the affine arrow that suggested F14-TLR1/2 as a stable flexible complex (**Figure 3A**; **Supplementary Figure S4**). This inference was validated by plotting elastic network maps (**Figure 3B**i; **Supplementary Figure S5**), which depict the motion stiffness of each protein complex. In these plots, each gray dot serves as a count for one spring connecting a pair of atoms (40). The limited number of gray dots in the elastic network of F14-TLR1/2 complex supports the presence of explicit flexible motion between the two interacting proteins in their complex form (**Figure 3B**i; **Supplementary Figure S5**).

On the other hand, covariance matrices highlight the relations among the pairs of residues in each MAg-TLR complex in terms of correlated (shown in red), uncorrelated (white) and anti-correlated (blue) residual motions (**Figure 3B**ii; **Supplementary Figure S6**) (40, 42). Here, the abundance of correlated motion contributing to molecular and atomistic dynamics throughout the F14-TLR1/2 complex highlighted the characteristic nature of F14 in forming complex with TLR1/2 when compared to the other five MAg-TLR complexes included in the analysis.

B-factor and deformability plots depict the relative amplitude of atomic displacements throughout the ligand-receptor complex under investigation (40, 42). Moderate degree of dispersion in the F14-TLR1/2 complex, along with the other five complexes, indicated a moderate level of rigidity (**Figure 3C**i; **Supplementary Figures S7**, **S8**). The F14-TLR1/2 complex showed a minor increase in amplitude within the range of 600-800 residues and the complex was otherwise observed to be stable throughout. Deformability plots further supported the conclusions drawn from B-factor analysis by illustrating the flexibility and stability of the F14-TLR1/2 complex. (**Figure 3C**ii; **Supplementary Figures S7**, **S8**).

The eigenvalue estimates the effect of each atomistic/residual deformation on the total protein motion (40). A low eigenvalue (1.684880e^-05^) for F14-TLR1/2 (**Figure 3D**; **Supplementary Figure S9**) implies requirement of very minimum energy to deform this complex and hence it exhibits excellent stability (42, 49). Variance is inversely proportional to the eigenvalue of a given complex and measures the percentage of fluctuation occurring within the complex (49). F14-TLR1/2 was found to achieve 80% variance within the first 7 modes counted out of the 20 modes, thus further confirming the greater stability of F14-TLR1/2 complex (**Figure 3E**; **Supplementary Figure S10**).

**Figure 4:**
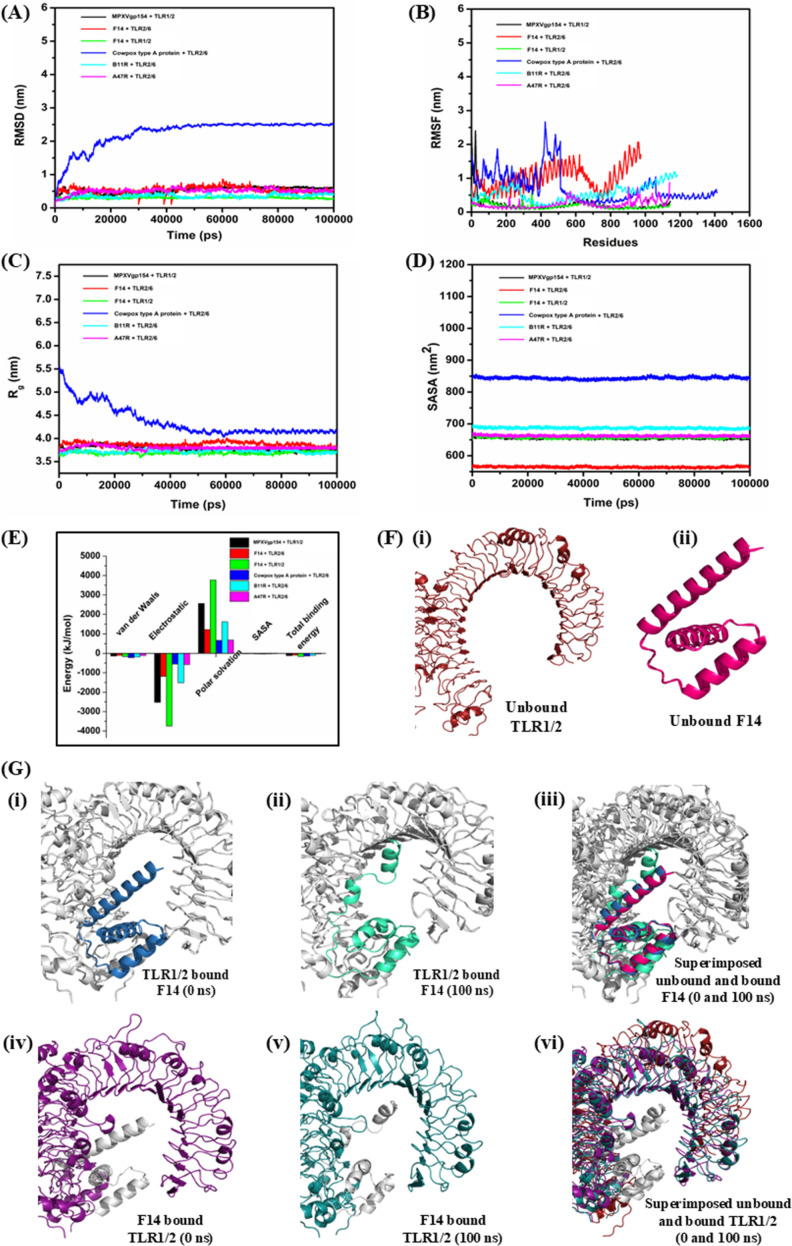
Comparative molecular dynamics simulation (MDS) trajectories depicting the biophysical and thermodynamic stability of protein-protein interactions in the F14-TLR1/2 complex, alongside other MAg-TLR complexes. **(A)** Root Mean Square Deviation (RMSD) plot, **(B)** Root Mean Square Fluctuation (RMSF) plot, **(C)** Radius of Gyration (R_g_) plot, **(D)** Solvent Accessible Surface Area (SASA) plot, **(E)** Binding free energy values. Different MAg-TLR complexes are shown in unique colors such as MPXVgp154 viral protein and TLR1/2 complex (black), F14 viral protein and TLR2/6 complex (red), F14 protein and TLR1/2 complex (green), Cowpox A-type inclusion viral protein and TLR2/6 complex (blue), B11R viral protein and TLR2/6 complex (cyan) and A47R viral protein and TLR2/6 complex (purple). **(F)** Binding of F14 induces conformational changes in TLR1/2. Changes in the conformation of (i) unbound TLR1/2 and (ii) unbound F14 before and after the formation of F14-TLR1/2 complex. **(G)** Conformational changes in F14 and TLR1/2 due to binding and forming a stable complex, shown as (i) TLR1/2 bound F14 (0 ns), (ii) TLR1/2 bound F14 (100 ns), and (iii) superimposed unbound and bound F14 (0 and 100 ns), (iv) F14 bound TLR1/2 (0 ns), (v) F14 bound TLR1/2 (100 ns), and (vi) superimposed unbound and bound TLR1/2 (0 and 100 ns). Conformational changes were observed for a period of 100 ns in molecular dynamics simulation platform.

These postulations were verified by comparative MDS trajectory analyses of F14-TLR1/2 complex along with the other five sorted MAg-TLR complexes viz. F14-TLR2/6, Cowpox A-type inclusion protein-TLR2/6, MPXVgp154-TLR1/2, B11R-TLR2/6 and A47R-TLR2/6 (**Figures 3A–E**). The comparative RMSD data affirmed conformational stability of F14-TLR1/2 complex, which exhibited the lowest deviation ranging under 0.5 nm with negligible oscillations throughout the 100 ns trajectory. This indicates high structural stability and strong binding of the complex compared to the other five complexes. (**Figure 4A**). Further, RMSF plots also reiterated the earlier inference on the stability of F14-TLR1/2 complex as minimal fluctuation of each amino acid contributed to the residual flexibility within a range of 0 to 1 nm. This suggests overall stability and the presence of helices and/or sheets in the flexible protein complex (**Figure 4B**).

The radius of gyration (Rg) is the mass-weighted root-mean-square distance of a collection of atoms from their common center of mass. It serves as a measure of the compactness of a protein complex, complementing other measurements such as RMSD and RMSF values (50). Herein, a lower R_g_ score of F14-TLR1/2 complex signified a greater stability. In fact, F14-TLR1/2 complex displayed a R_g_ score within the stable range of 3.5 to 4.0 nm, in comparison to the other five MAg-TLR complexes. This further supports the structural stability of the complex formed by the binding of F14 to TLR1/2 (**Figure 4C**). Furthermore, F14-TLR1/2 complex had a solvent accessible surface area (SASA) value of above 650 nm^2^ throughout the 100 ns dynamics without any major fluctuation thus indicating that the complex is potentially a stable one (**Figure 4D**).

We also examined the thermodynamic stability of F14-TLR1/2 complex by computing the overall binding free energy. The data revealed that binding energy of F14 is maximized due to the electrostatic energy that reached beyond -3500 kJ mol^-1^, and is most opposed by the polar solvation energy of nearly 3700 kJ mol^-1^ (**Figure 4E**). Comparative binding energy analyses showed the involvement of a low binding free energy for F14-TLR1/2 complex in stabilizing the receptor and ligand in the complex thus validating the claims that F14 is a putative ligand of TLR1/2.

Lastly, post MD simulation conformational changes in F14 and TLR1/2 were analyzed (**Figures 4F, G**; **Supplementary Figure S11**). Changes in the conformation of a receptor protein due to binding of a ligand, following protein-protein interactions, are widely considered as the most acceptable evidence to judge the candidature of two proteins as interacting partners (40). Here, clear evidence of conformational changes in F14 (**Figures 4F**i, **G**i–iii) as well as in TLR1/2 (**Figures 4F**ii, **G**iv–vi) during the formation of initial complex to the final stable F14-TLR1/2 complex collectively supported F14 as a putative ligand of TLR1/2.

### Characterization of F14 protein and in-silico cloning

**Figure 5:**
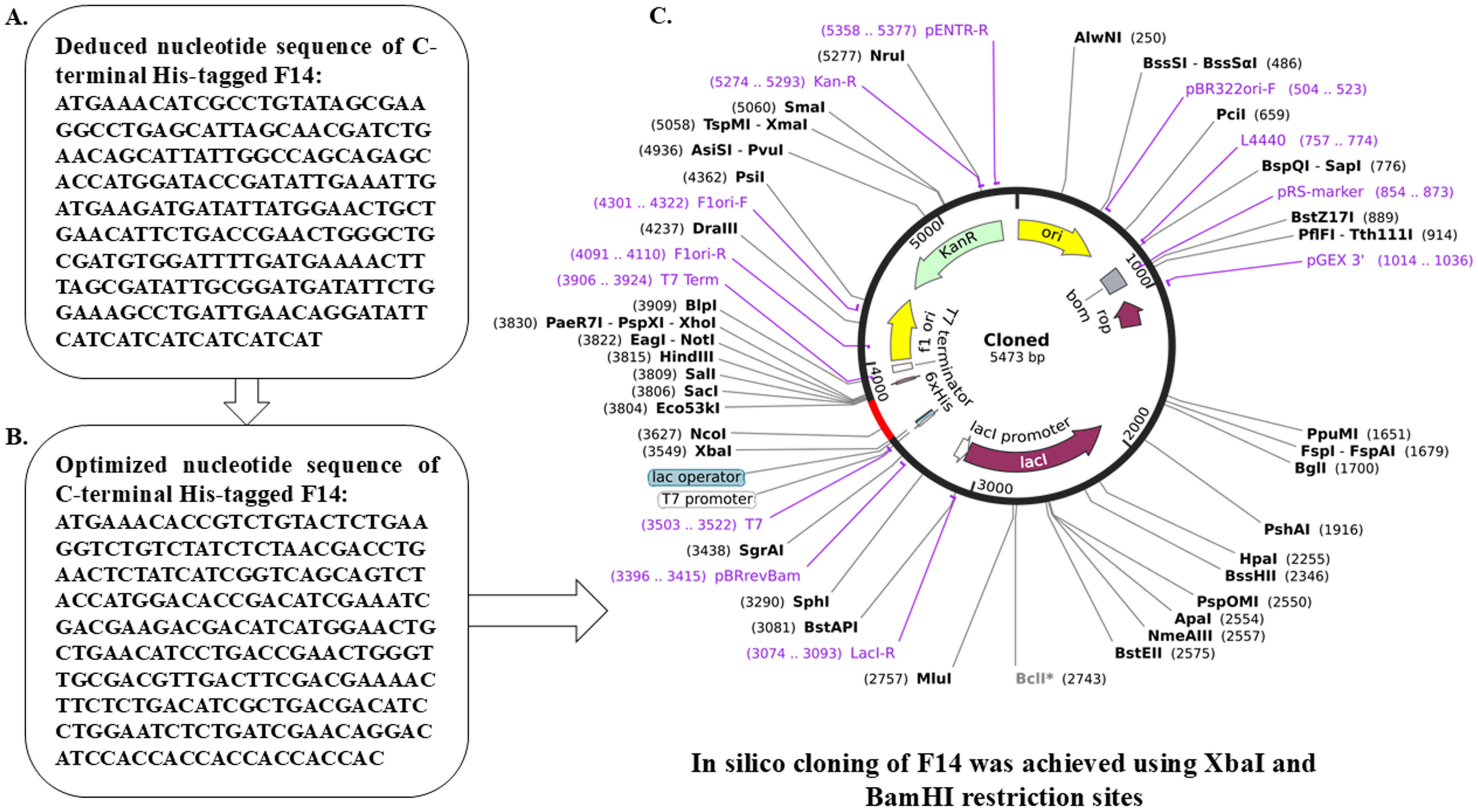
*In silico* cloning within *E. coli* to obtain recombinant F14 protein. **(A)** Deduced nucleotide sequence of Monkeypox F14 protein. **(B)** Optimized cDNA sequence of F14 with C-terminal His tag. **(C)** Map of cloned F14 cDNA within the expression vector for recombinant protein production.

After confirming the functional identity of Mpox F14 protein, various biochemical characters such as isoelectric pH (pI:3.4), aliphatic index (112.19), GRAVY (-0.223) and pattern of post-translational modification were computed. Importantly, this protein appears to be non-allergenic (**Supplementary Figure S11**; **Supplementary Tables S7**, **S8**). The ProtPARAM server was used to determine the physicochemical characteristics of the F14 antigen. This 73 amino-acid protein was reported to be of 8.28 kDa in molecular weight, consisting of 1137 atoms in total, with an isoelectric pH of 3.4 and a sub-cellular localization in the host-cell cytoplasm. The aliphatic index was determined to be 112.19 and it acquired a GRAVY score of -0.223. Further, the Active Site Prediction online server identified three types of active sites to appear on the viral antigen F14 (**Supplementary Table S9**), namely N-glycosylation sites (1 site found), casein kinase II phosphorylation sites (3 sites found) and N-myristoylation sites (2 sites found). Gene sequence was deduced and cDNA encoding F14 was cloned in an expression vector pET30ax with 6 histidine (His) tags in the C-terminus for recombinant production in *Escherichia coli* (**Figure 5**).

## Discussion

Immunopathogenesis in Orthopoxvirus infections is a decisive factor that determines severity of the disease and clinical outcome. In the case of Mpox, the physical manifestations of the disease are directly connected with the cytokine responses (51). In fact, immunomodulatory proteins of Vaccinia virus (VV) have been shown to downregulate components of the TLR signaling pathway. For example, A46R interferes with MAP kinase and NF-κB activation by associating with TIR adaptors, A52R targets TRAF6 and IRAK2 to inhibit TLR-mediated NF-κB activation while upregulating IL-10 production, and K7 targets DDX3, which is essential for the induction of IFN-β (33, 52, 53). Recently, a comparative cytokine profiling has been performed by using sera from the patients with active Mpox infection and healthy individuals (51). The elevated levels of key pro-inflammatory cytokines like IL-6, IL-1β and IL-8 in these patients with severe grades of the disease point toward the involvement of NF-κB signaling in Mpox infection. As TLRs are known to be the primary PRRs responsible for sensing the invading pathogens including viruses, this data raises an important question about the significance of cell-surface TLRs in inducing initial inflammatory signals upon Mpox infection (44, 54).

In this context, TLR2, in a heterodimeric combination with TLR1 or TLR6, and TLR4 are key cell-surface TLRs responsible for recognizing the viruses. These TLRs interact with viral antigens, which serve as PAMPs (55). Viral antigen-TLR interactions trigger the production of inflammatory cytokines and interferons. Of note, the induction of type I IFN and other inflammatory cytokines through MyD88-dependent TLR2 signaling has been reported following the recognition of VV ligands (55, 56). VV-based *in-vitro* and *in-vivo* studies using TLR2^-/-^ and MyD88^-/-^ mice have revealed the importance of TLR2 and MyD88 in the recognition of Orthopoxvirus (57). It is also noteworthy that the protein sequences and structures of Orthopoxvirus members share a high degree of similarity (58). Additionally, cytokine profiling of Mpox patients with varying disease severities provided evidence of M1 macrophage polarization and inflammatory cytokine responses. Together, these findings, along with our advanced bioinformatics analysis, provide a pointer toward potential molecular mechanisms underlying viral immunopathogenesis.

The current study was initiated by examining the sequences of surface and intracellular antigens of the Mpox virus using public databases (e.g., UniProt) and relevant literature. A total of 174 protein sequences were identified, which were then modeled to generate their corresponding 3D crystal structures and followed by analyses of their stereochemical properties. Of the 174 viral protein sequences, 105 were successfully modeled using homology modeling. Additionally, we retrieved the crystal structures of all human cell-surface TLRs from the PDB. We then assessed the potential of these viral antigens to act as ligands for TLR1/2, TLR4, TLR5, and TLR2/6 by performing in-silico protein-protein interaction analyses through molecular docking, followed by a comparative evaluation of the biophysical interactions. These results revealed that 79 of the modeled protein antigens could form complexes with human TLRs, exhibiting varying degrees of affinity (**Supplementary Table S2**).

After a thorough investigation of binding affinity for all the MAgs based on binding free energy (-ΔG), we identified five proteins—F14, A47R, Cowpox A-type inclusion protein, MPXVgp154 and B11R as the strongest interacting partners of human TLR1/2 and TLR2/6 heterodimers (**Figure 1**). We further analyzed the most stable and strongly bound MAg-TLR complexes by investigating the biophysical interactions between the MAgs and human TLRs (**Table 1**; **Supplementary Tables S4**, **S5**). Among the six stable Mpox-TLR complexes formed by the interactions of F14, A47R, Cowpox A-type inclusion protein, MPXVgp154, and B11R with human TLR1/2 and TLR2/6 heterodimers, the F14-TLR1/2 complex was identified as the most stable, based on both binding free energy and the extent of biophysical interactions (**Figures 1C, D**). Our *in silico* data suggest that F14 plays a crucial role in mediating the pathogenesis of Mpox infection by acting as a ligand for cell-surface TLR1/2, thereby eliciting inflammatory signals during the infection.

F14 is a 73 amino acid Mpox protein with a molecular weight of 8.281 kDa and is found in the cytoplasm of infected host cells. Previous studies have explored the potential role of F14 protein in mediating the immunopathogenesis in various Orthopoxvirus infections (59). For instance, F14 from VV was shown to mimic the transactivation domain of the NF-κB p65 sub-unit, selectively inhibiting the expression of NF-κB-regulated genes (59). In our study, F14 was found to occupy the extracellular binding pocket of both TLR1/2 and TLR2/6 (**Figure 1**). However, comparison of binding strength and affinity indicated a notably stronger interaction of F14 with the TLR1/2 heterodimer, suggesting that F14 is a potent ligand for TLR1/2. This observation prompted us to investigate the mechanistic insights on the strong affinity of F14 for the TLR1/2 heterodimer and its ability to form a thermodynamically stable complex.

To investigate this further, we conducted MD trajectory analyses. Comparative NMA data revealed significant deformation and flexibility of both F14 and the TLR1/2 heterodimer when in complex (**Figure 3**). MD simulations supported these findings, confirming that the F14-TLR1/2 complex exhibits superior structural and conformational stability compared to other complexes (**Figure 3**). We also examined the role of individual amino acids in F14 involved in the protein-protein interactions by introducing mutations within F14 (**Figure 2**; **Supplementary Table S6**). Notably, a mutation at the GLU70 residue at the amino terminus of F14 significantly reduced its binding affinity to TLR1/2, indicating that GLU70 is a critical residue for maintaining the bound-state topology of the F14-TLR1/2 complex (**Figure 2B**; **Supplementary Table S6**).

Overall, these data suggest that F14 is a potential ligand for the human TLR1/2 dimer, likely triggering TLR1/2-driven pro-inflammatory responses in the infected host. These insights highlight a promising avenue for effectively targeting the immunopathogenesis of Mpox. Although Mpox infection has been shown to subside with anti-smallpox therapeutics, the evolutionary tendency of the virus suggests significant changes in its immunomodulatory strategies and a potential increase in pathogenicity (60). Numerous reports indicate rising gene copy numbers of the Mpox virus, the absence of classical symptoms, and the emergence of symptoms differing from those observed in previous outbreaks (61, 62). Notably, there has been an increase in atypical symptoms, such as anogenital lesions and a lower incidence of rashes. These observations underscore the urgent need for developing Mpox-specific treatment strategies to combat this disease, which could be accomplished by targeting the Mpox-TLR interactions through developing vaccine and/or immunotherapeutics.

In this context, F14 protein could represent a promising candidate. Firstly, Mpox F14 shares a high degree of homology with other members of Poxviridae family such as vaccinia, cowpox, variola, raccoonpox, skunkpox virus and others. (**Supplementary Figure S12**). Secondly, Mpox F14 contains a negatively charged dipeptide (D62/63) that has been shown to mimic p65, dampening NF-κB-dependent transcription of antiviral genes and thereby increasing viral load (63) and this effect was reversed by targeting F14 at the protein (64). Our present study in line with these recent observations suggest that F14 could be a key target for developing peptide/mRNA vaccine to counteract Mpox infection in the future. In particular, a peptide-based vaccine could be developed in combination with a novel oil-in-water (O/W) emulsion-based vaccine adjuvant, which is currently undergoing clinical trial (65).

## Conclusion

Various approaches have been used recently to identify the vaccine candidates and drug targets for Mpox that include designing of multi-epitope vaccines by bioinformatics approach (66–68), studying the human-monkeypox virus interactome to identify potential drug targets (69) and a combination of recombinant structural proteins viz. A29L, M1R, A35R, and B6R as vaccine adjuvants for restoring IFN-γ response in animal model (70). In this conext, our in-silico study highlights Mpox F14 antigen as one of the critical mediators of host-virus interactions and pathogenesis in Mpox-infected subjects. Therefore, F14 could serve as a promising target for developing the vaccine in the near future, although experimental validation through pre-clinical and clinical trials is necessary to confirm its potential.

## Data Availability

The original contributions presented in the study are included in the article/**Supplementary Material**. Further inquiries can be directed to the corresponding authors.
