# Supplementary material for: In silico evidence of monkeypox F14 as a ligand for the human TLR1/2 dimer
Source: Front Immunol. 2025 Mar 17;16:1544443. doi: 10.3389/fimmu.2025.1544443 (PMC11955672; doi:10.3389/fimmu.2025.1544443)
Supplement: Supplementary file 1 [file DataSheet1.pdf]

Supplementary Table S1. Mining of immunoregulatory Mpox antigens from the UniProt database.

Sl no	Protein names	Accession number	Gene name	Length	Function	Reference
1.	Cell surface-binding protein	A0A7H0DN92	OPG105	304	Binds to chondroitin sulphate on the cell surface to provide virion attachment to target cell	UniProt Accession ID: UP000516359
2.	J2L (J2R)	P0DTN0	OPG002	349	Inhibits host immune defence by binding to host TNF and various chemokines in the extracellular space; binds host CC and CXC chemokines	
3.	DNA-directed RNA polymerase	A0A7H0DNC3	OPG151	1164	Responsible for the transcription of early, intermediate and late genes	
4.	Major core protein OPG136 (4a) precursor	A0A7H0DNA8	OPG136	891	Major component of the virion core that undergoes proteolytic processing during the immature virion (IV) to mature virion (MV) transition	
5.	A10L	A0A0F6N7H7	A10L	100	Required for an early step in virion morphogenesis	
6.	Scaffold protein D13	Q3I8Q4	E13L	551	Transitory spherical honeycomb lattice formed by D13 provides curvature and rigidity to the convex membrane of crescent and immature virions	
7.	Protein L2	A0A7H0DN68	OPG096	92	Formation and elongation of crescent-shaped membrane precursors of immature virions in cytoplasmic factories	
8.	Metalloendopeptidase	A0A7H0DN57	OPG085	591	Involved in maturation of some viral proteins by processing them preferentially at Ala-Gly-|-Ser/Thr/Lys motifs	
9.	Protein OPG079 (I3)	A0A7H0DN51	OPG079	269	Essential role in viral DNA replication	
10.	Virion membrane protein OPG147 (A21)	A0A7H0DNB9	OPG147	115	Envelope protein part of the entry-fusion complex responsible for the virus membrane fusion with host cell membrane during virus entry; role in cell-cell fusion	
11.	Core protein OPG142 (A15)	A0A7H0DNB4	OPG142	94	Part of a complex which participates in the formation of virosomes and the incorporation of virosomal contents into nascent immature virions	
12.	A15L	Q5IXQ0	A15L	90	Major component of mature virion; essential for membrane biogenesis	

13.	25 kDa core protein A12L	Q5IXQ2	A13L	190	Essential for the formation of a structurally normal core	
14.	Protein A11	Q5IXQ3	A12R	318	Required for viral crescent formation early during virus morphogenesis	
15.	Major core protein OPG129 (4b)	M1KJ27	OPG129	644	Essential for the formation of a structurally normal core	
16.	Entry-fusion complex protein OPG094 (L5)	A0A7H0DN71	OPG099	128	Component of the entry-fusion complex which mediates entry of the virion core into the host cytoplasm	
17.	Core protein VP8	M1L511	OPG098	251	Major core structural protein	
18.	MPXV-COP-073 (MPXVgp079)	A0A7G8C328	G10R	340	An entry/fusion complex component	
19.	Protein G3	Q5IXV4	G2L	111	Viral entry into the host cell	
20.	Protein OPG078 (I2)	M1L4Z5	OPG078	73	Late protein which probably plays a role in virus entry into the host cell	
21.	Serine/threonine-protein kinase 2	A0A7H0DN27	OPG054	439	Essential serine-protein kinase involved in the early stage of virion morphogenesis	
22.	Bcl-2-like protein IFN-beta inhibitor (D14L)	V9NLA0	D14L	216	Involved in many recognition processes, including the binding of several complement factors to fragments C3b and C4b	
23.	Viral core cysteine proteinase	A0A0F6N6K4	MPXV-Congo_8-061	423	Proteolysis	
24.	Cowpox A-type inclusion protein	A0A0F6N6S2	MPXV-Congo_8-131	512	Fusion of virus membrane with host plasma membrane	
25.	Putative type-I membrane glycoprotein	A0A0F6N6S9	MPXV-CAM1990_02-145, MPXV-Congo_8-146, MPXV-GAB1988_001-	196	Unknown	

			146, MPXV-Ikubi-145			
26.	Ankyrin-like protein	A0A7H0DNH0	OPG003	588	May be involved in virus-host protein interaction through the ankyrin repeats and PRANC regions	
27.	CPXV205 protein	A0A0F6N791	MPXV-Congo_8-163	109	Unknown	
28.	B6R (MPXVgp167)	A0A2L0ARH9	B6R	317	EEV membrane glycoproteinn	
29.	A14L (MPXVgp124)	V9NS49	A14L	70	Essential for the encapsidation of DNA into immature virions (IV) and the subsequent maturation of IV into mature virions (MV)	
30.	Anti-apoptotic Bcl-2-like protein (MPXVgp023)	M1LKP0	P1L	117	Unknown	
31.	A-type inclusion body protein (A27L)	O02685	A27L	696	Viral process	
32.	MPXVgp092	A0A7H0DN79	OPG107	189	Fusion of virus membrane with host membrane;<br>also plays a role in cell-cell fusion (syncytium formation)	
33.	L1R (MPXVgp085)	Q3I7N2	L1R	152	Unknown	
34.	Ankyrin-like protein (J1R) (J3L)	Q3I8G6	J3L	587	Unknown	
35.	B21R	Q3I8H3	B21R	1879	Unknown	
36.	B19R	Q3I8H5	B19R	357	Unknown	
37.	B13R	Q3I8I4	B13R	149	Unknown	
38.	B11R	Q3I8I6	B11R	282	ATP binding and Signal Transduction	
39.	6 kDa intracellular viral protein (B10R)	Q3I8I8	B10R	221	Unknown	
40.	21kDa protein	Q3I8J0	B8R	182	Unknown	
41.	Ankyrin-like protein (B5R)	Q3I8J3	B5R	561	Unknown	

42.	Protein A51	A0A7H0DNE7	OPG181	334	Unknown	
43.	A47R	Q3I8K3	A47R	240	TIR-like protein	
44.	A41L	Q3I8K9	A41L	221	Unknown	
45.	A40L	Q3I8L0	A40L	277	Promotes, when overexpressed, the influx of extracellular Ca2+, leading to membrane permeability and host cell necrosis	
46.	CPXV173 protein	Q3I8L2	A39R	268	Unknown	
47.	A38R	Q3I8L3	A38R	212	Unknown	
48.	MHC class II antigen presentatio n inhibitor	Q3I8L4	A37R	176	Unknown	
49.	A33R	Q3I8L8	A33R	145	Unknown	
50.	A32L	Q3I8L9	A32L	77	Required for the association between the dense viroplasm and the viral membranes to form the mature virion (MV)	
51.	A18L	V9NVD7	A18L	204	Unknown	
52.	39 kDa core protein (A5L)	V9NU24	A5L	281	Component of the virion core; participates in virion assembly	
53.	H3L	V9NPG6	H3L	324	Symbiont entry into host cell	
54.	ER-localized membrane protein	Q3I8V8	F7R	273	Unknown	
55.	Protein E6	A0A7H0DN41	OPG068	567	Plays an essential role for maintaining proper localization of the seven-protein complex and the viroplasm during assembly	
56.	EEV maturation protein	Q3I8X2	C18L	635	Microtubule-dependent intracellular transport of viral material towards cell periphery	
57.	Protein F11	Q3I8X3	C17L	354	Stimulates increases in peripheral microtubule dynamics and may increase the motility of the infected cells, contributing to cell-to-cell spread of the virus	
58.	36 kDa major membrane protein	Q3I8X9	C11L	343	Plays a role in the spread of virus to neighboring cells ex vivo	
59.	Caspase-9	Q3I8Y3	C7L	219	Regulation of apoptotic process	
60.	B15R-like protein	Q3I8Y4	C6R	149	Virus-mediated perturbation of host defense response	
61.	Monoglyce ride lipase	A0A7H0DN16	OPG043	276	Unknown	
62.	NFkB inhibitor	Q3I8Y9	O2L	220	Unknown	

63.	Ankyrin-like protein	Q3I919	D1L (N2R)	437	Unknown	
64.	Bifunctional IL-1-beta-inhibitor	Q3I934	B14R	326	Interleukin-1 binding	
65.	Ankyrin-like protein (B7R)	Q3I942	B7R	176	Unknown	
66.	MPXVgp154	A0A7H0DNE1	OPG173	74	Inhibits the initiation of cap-dependent and cap-independent translation; affects the outcome of infection by decreasing recruitment of inflammatory leukocytes and reducing the memory CD8+ T-cell response	
67.	Assembly protein G7	Q49QI0	VACV_BRZ_SERRO2_083	371	Late protein which is a part of a large complex required for early virion morphogenesis. This complex participates in the formation of virosomes and the incorporation of virosomal contents into nascent immature virions	
68.	Kelch-like protein	Q3I9I1	C9L	492	Unknown	
69.	L5L (MPXVgp089)	V9NP69	L5L	133	Unknown	
70.	B16R	Q5CA82	B16R	352	IFN-alpha/beta-receptor-like secreted glycoprotein	
71.	B12R (MPXVgp173)	Q5IXK4	B12R	344	Unknown	
72.	B9R (MPXVgp170)	Q5IXK7	B9R	267	Cytokine receptor activity	
73.	Profilin	Q8V4T7	OPG171	133	More likely to influence phosphoinositide metabolism than actin assembly	
74.	EEV glycoprotein (MPXVgp146)	Q5IXM9	A36R	168	Membrane protein	
75.	A30L (MPXVgp140)	Q5IXN6	A30L	146	Envelope protein required for virus entry into host cell and for cell-cell fusion (syncytium formation)	
76.	A17L (MPXVgp127)	Q5IXP7	OPG143	377	Envelope protein part of the entry-fusion complex responsible for the virus membrane fusion with host cell membrane during virus entry. Also plays a role in cell-cell fusion	
77.	A15.5L	Q5IXP9	A15.5L	53	Membrane protein	

78.	A7L (MPXVgp117)	M1L535	A7L	372	Plays an essential role in immature virion (IV) to mature virion (MV) transition	
79.	A3L (MPXVgp113)	Q5IXR2	OPG128	77	Late protein which probably participates in disulfide bond formation by functioning as a thiol-disulfide transfer protein between membrane-associated OPG072 and OPG08. The complete pathway for formation of disulfide bonds in intracellular virion membrane proteins sequentially involves oxidation of OPG072, OPG128 and OPG08	
80.	E2L (MPXVgp099)	Q5IXS6	E2L	146	Late protein which is part of a large complex required for early virion morphogenesis. This complex participates in the formation of virosomes and the incorporation of virosomal contents into nascent immature virions	
81.	Internal virion protein (M3L) (MPXVgp082)	Q5IXU3	M3L	344	Internal virion protein	
82.	IMV membrane protein (M1R) (MPXVgp080)	Q5IXU5	M1R	250	Viral envelope protein; unknown	
83.	IMV protein VP13 (MPXVgp066)	Q5IXV9	I5L	79	Envelope protein	
84.	Core protein E11 (MPXVgp059)	A0A7H0DN46	OPG073	129	Unknown	
85.	MPXV-COP-049 (MPXVgp055)	Q5IXX0	F6R	166	Transferase activity	
86.	Core phosphoprotein F17 (MPXVgp049)	Q5IXX6	C23R	101	DNA-binding	
87.	Protein F16	A0A7H0DN34	OPG061	231	Unknown	
88.	Protein F15	A0A7H0DN33	OPG060	158	Unknown	

89.	Protein F14	A0A7H0DN31	OPG058	73	Unknown	
90.	Envelope protein F13 (MPXVgp0 45)	A0A7H0DN30	OPG057	372	Major envelope protein that plays a role in the biogenesis of the viral double membrane and in egress of virus from the host cell. Produces the wrapped form of virus that is required for cell-to-cell spread. Acts as a lipase with broad specificity including phospholipase C, phospholipase A, and triacylglycerol lipase activities	
91.	Envelope protein F13 (MPXVgp0 30)	Q5IXZ5	C4L	424	Viral envelope protein; catalytic activity	
92.	N3R	Q5QC67	N3R	176	TNF-receptor-like protein	
93.	Ankyrin-like protein (C1L)	Q5QC71	C1L	284	Unknown	
94.	Ankyrin-like protein (O1L)	Q5QC73	O1L	442	Unknown	
95.	Alpha-amanitin target	Q5QC74	P2L	177	Putative TLR signaling inhibitor alpha	
96.	D19L	Q5QC76	D19L	214	Putative TLR signaling inhibitor	
97.	D13L	Q5QC82	D13L	315	Receptor	
98.	BTB domain of kelch-like protein (D12L)	Q5QC83	D12L	206	Unknown	
99.	D10L	A0A7H0DN02	OPG027 (D10L)	150	Inhibits antiviral activity induced by type I interferons. Does not block signal transduction of IFN, but is important to counteract the host antiviral state induced by a pre-treatment with IFN	
100	Ankyrin-like protein (D9L)	Q5QC86	D9L	630	Transcription cis-regulatory region binding	
101	D8L	Q5QC87	D8L	64	Unknown	
102	Ankyrin-like protein (D7L)	Q5QC88	D7L	660	Unknown	
103	Interleukin-18-binding protein D6L (MPXVgp0 09)	A0A7H0DMZ 8	OPG022 (D6L)	126	Soluble IL18-binding protein that may modulate the host antiviral response	
104	CC-type chemokine	V9NKZ8	J3R	252	Chemokine-binding activity	

	binding protein					
105	A35R	Q80KX2	A35R	181	Carbohydrate-binding	
106	B2R	Q8AZ47	HA	313	Bifunctional hemagglutinin/type-I membrane glycoprotein	
107	14 kDa protein (A29L)	Q9YN60	A29L	110	Fusion of virus membrane with host membrane	

Supplementary Table S2. Screening of TLR-binding Mpox antigens through molecular docking of Mpox antigens with human cell-surface toll-like receptors (TLRs).

Sl no.	Name of viral antigen	Molecular docking scores with different human cell-surface TLRs			
		TLR1/2	TLR2/6	TLR4	TLR5
1.	A3L	-619.0	-806.8	-806.8	-
2.	A7L	-389.4	-644.3	-392.5	-
3.	A15.5L	-847.5	-974.2	-651.9	-
4.	Alpha-amanitin target protein	-626.3	-1002.7	-559.8	-
5.	Ankyrin like protein 1	-676.0	-555.6	-236.8	-
6.	Bifunctional IL-1β inhibitor	-	-	-339.1	-
7.	A11	-769.0	-902.6	-231.5	-458.1
8.	A10L	-621.0	-701.9	-854.4	-530.8
9.	A12L	-	-131.2	-152.0	-474.4
10.	A15	-1023.2	-925.3	-544.3	-345.0
11.	A15L	-891.6	-909.1	-976.9	-500.1
12.	A21	-908.1	-839.4	-799.2	-603.0
13.	A40L	-379.6	-445.2	-347.4	-333.4
14.	A41L	-496.3	-548.0	-386.5	-441.6
15.*	A47R	-556.3	-1217.1	-273.6	-496.2
16.	A51	-384.2	-548.0	-	-239.4
17.	Ankyrin-like protein 2	-1050.5	-959.0	-569.0	-694.8
18.	Ankyrin-like protein 3	-451.7	-490.3	-427.6	-875.5
19.	B15R	-643.4	-1110.5	-175.4	-394.9

20.	CPXV173	-528.9	-872.1	-435.3	-
21.	D13	-563.7	-530.3	-243.1	-
22.	I3	-826.8	-917.9	-357.2	-423.8
23.	D19L	-513.8	-	-464.0	-
24.	E2L	-675.3	-838.6	-	-
25.	E11L	-621.3	-943.6	-	-
26.	ER-localized membrane protein	-528.3	-	-576.4	-
27.	F13	-549.9	-	-	-
28.*	F14	-1965.0	-1519.7	-224.1	-
29.	F15	-617.6	-	-730.1	-
30.	F16	-942.2	-	-788.7	-
31.	F17	-485.7	-	-988.1	-
32.	IMV membrane protein target	-717.8	-	-581.1	-
33.	IMV membrane protein	-553.4	-	-372.8	-
34.	Bifunctional 21kDa protein	-454.2	-696.1	-266.6	-
35.	Core protein 4a	-581.1	-	-	-
36.	MHC class-II antigen presentation protein	-489.8	-	-	-
37.	MPXVcop049	-564.5	-	-	-
38.	MPXVcop073	-	-	-	-
39.	MPXVgp092	-362.3	-365.0	-457.0	-
40.*	MPXVgp154	-1226.7	-975.6	-628.2	-
41.	6kDa protein	-391.0	-404.8	-484.9	-453.1
42.	21kDa protein	-488.9	-	-404.3	-
43.	B6R	-	-	-113.5	-
44.*	B11R	-476.2	-1215.3	-458.6	-355.1
45.	B13R	-364.3	-976.7	-308.7	-
46.	Bcl2-like protein	-	-141.6	-	-
47.	D6L	-686.4	-782.4	-535.3	-
48.	Gp092	-686.4	-	-	-
49.	Metallopeptidase	-550.7	-587.9	-502.8	-

50.	D8L	-	-785.3	-1112.5	-826.9
51.	A14L	-178.9	-429.2	-484.7	-528.4
52.	A32L	-735.5	-837.8	-886.8	-
53.	Anti-apoptotic Bcl2-like protein	-1022.0	-940.6	-	-396.6
54.	BTB domain of Kelch-like protein	-	-1006.1	-260.6	-267.1
55.	CC-type chemokine binding protein	-664.1	-638.7	-161.6	-369.0
56.	Cell surface binding protein	-430.1	-718.3	-	-472.9
57.	CPXV205	-827.9	-961.3	-161.6	-207.4
58.	D10L	-717.8	-1107.8	-688.1	-550.8
59.	G3	-374.1	-846.0	-627.0	-395.5
60.	G7	-	-382.1	-256.7	-489.9
61.	I2	-955.0	-885.8	-724.3	-441.1
62.	H3L (IMV heparin-binding surface protein)	-445.1	-1024.4	-275.8	-200.8
63.	IMV protein	-429.4	-720.9	-322.2	-483.7
64.	IMV surface protein	-721.9	-780.4	-656.9	-394.8
65.	J2L	-134.9	-136.8	-318.6	-312.4
66.	L5	-	-194.4	-294.2	-357.6
67.	Type-1 glycoprotein	-531.3	-	-	-
68.	B19R	-598.7	-987.5	-	-
69.	Viral core cysteine protease	-486.3	-561.6	-566.9	-
70.	VP8	-564.7	-907.3	-	-
71.	39kDa protein	-174.7	-390.9	-126.5	-169.7
72.	B2R	-	-545.0	-	-
73.	Bcl-2 like protein	-	-940.6	-	-
74.	D12L	-	-	-	-
75.	TNF-receptor-like protein	-598.9	-	-	-
76.	VP13	-663.1	-	-	-
77.*	Cowpox type-A inclusion protein	-	-1389.5	-	-
78.	Putative type-1 membrane glycoprotein	-	-459.5	-524.8	-

79.	Serine threonine protein-kinase	-	-390.9	-591.8	-

*Rows with grey shade represent significant binding between Mpox antigens and human TLRs resulting in the formation of stable complexes, which were analyzed further.

Supplementary Table S3. Stereochemical qualities of the 5 selected TLR-binding Mpox proteins.

Name of Protein	Overall Quality Factor from ERRAT plots	Ramachandran Plot Values (in %)			
		Within most favoured regions	Within additionally allowed regions	Within generously allowed regions	Within disallowed regions
**F14**	100.000	91.2	8.8	0.0	0.0
**Cowpox A type inclusion protein**	91.540	90.2	9.3	0.4	0.0
**MPXVgp154**	98.182	88.6	10.0	1.4	0.0
**B11R**	95.437	90.9	8.7	0.4	0.0
**A47R**	98.253	90.7	7.9	0.0	1.3

Supplementary Table S4. Analysis of biophysical interactions between Cowpox A-type inclusion protein - TLR2/6 and MPXVgp154 - TLR1/2 complexes.

Cowpox A-type inclusion protein - TLR2/6 complex			MPXVgp154 - TLR1/2 complex		
Residue of Cowpox A -type inclusio n protein	Residue of TLR	Distance (Å)	Residue of MPXVgp154 protein	Residue of TLR	Distance (Å)
**Hydrogen bonds**					
ASP382	ARG1115	1.97	GLN43	GLN438	1.84
ASP380	ARG1207	1.74	LYS51	TYR531	2.87
ASP375	GLN1252	2.20	LYS54	TYR488	2.26
ASP368	ASN1281	1.72	LYS58	SER513	2.59
ASP372	ASN1310	1.95	LYS51	GLU529	2.83
ASP372	TYR1312	1.72	VAL39	GLN438	2.13
ASP378	HIS1137	2.65	ILE61	ASN514	2.19
			ASP62	ARG534	2.76
			ASN59	ARG534	1.89
			ASP62	ARG556	2.80
			ASP68	SER1131	1.61

			ASP68	GLY1132	1.94
			LYS51	TYR531	2.49
			LYS54	ASP467	2.36
			LYS58	SER492	2.10
			GLU57	ARG443	2.55
			GLU57	ARG443	2.66
			ILE61	ARG534	2.78
			ASP65	SER1076	2.76
			GLU64	LYS1080	2.43
			ASP68	PRO1102	2.73
**Hydrophobic interactions**					
			ALA46	TYR412	4.69
			VAL47	TYR488	4.47
			ALA50	TYR488	5.18
			LYS51	TYR488	4.97
**Electrostatic interactions**					
ASP382	ARG1115	4.56	ASP38	LYS387	5.12
ASP380	ARG1207	4.72	GLU49	ARG388	5.09
ASP378	PHE1158	4.25 (pi-anion)	ASP62	ARG556	4.09
**Salt bridges**					
ASP382	ARG1115	1.79	LYS51	GLU529	2.83
ASP380	ARG1207	1.83	LYS51	GLU529	1.84
ASP371	LYS1225	1.69	LYS54	ASP467	1.66
ASP368	LYS1225	3.18	LYS58	ASP511	1.81
ASP382	ARG1115	1.79	GLU49	ARG388	3.21
ASP380	ARG1207	1.83	GLU49	ARG388	1.82
ASP371	LYS1225	1.69	GLU57	ARG443	1.76
ASP368	LYS1225	3.18	GLU57	LYS470	2.18
			ASP62	ARG534	1.85
			ASP66	LYS536	1.88
			GLU64	LYS1080	2.02

Supplementary Table S5. Analysis of biophysical interactions between B11R-TLR1/2 and A47R+TLR2/6 complexes.

**B11R-TLR1/2 complex**			**A47R+TLR2/6 complex**		
**Residue of B11R protein**	**Residue of TLR**	**Distance (Å)**	**Residue of A47R protein**	**Residue of TLR**	**Distance (Å)**
**Hydrogen bonds**					
SER23	PHE116 9	2.75	GLN923	SER158	1.77
SER23	PHE116 9	1.85	GLU940	ASN232	1.84
LYS111	LYS678	2.67	VAL976	LYS321	1.75
LYS111	SER680	2.85	ASN977	LYS321	2.83
LYS111	SER680	2.73	ASN1008	LYS396	2.02
LYS111	LYS678	2.89	ASP1018	ARG460	1.89
SER113	ARG742	2.81	ASP1018	ARG482	2.94
SER113	ASN723	2.06	ASP1018	ARG482	1.78
SER113	ARG742	2.95	GLN1023	LYS535	2.86

GLY213	HIS705	2.71	SER907	LYS889	1.94
GLU247	SER706	2.03	ASP958	LEU896	2.85
GLU247	SER706	1.86	LYS912	LEU899	2.10
PHE212	ASN724	2.86	GLN923	ASP156	1.74
ASN249	ASN726	2.14	GLN924	MET133	2.15
GLU114	ARG742	1.90	GLN924	ASP134	3.03
GLU114	ARG764	1.99	THR978	ARG290	2.90
GLN281	GLN766	2.21	SER981	ASP260	1.64
GLN281	GLN766	2.01	TYR989	ASN35	2.78
GLU221	TYR108 3	2.88	LYS1022	HIS505	2.36
ASN220	SER1138	1.85	GLN923	SER158	2.79
ASN220	ASN115 8	2.43	PRO979	ARG290	2.48
THR20	LYS1171	2.75	ASN977	LYS321	2.73
ASN25	LEU117 8	2.61	GLN1023	LYS535	2.61
THR20	GLU117 0	2.24	ASP958	VAL894	2.78
HIS51	VAL117 6	2.69	LYS912	LEU899	2.56
ASP112	ASN722	3.02	SER981	ASP260	2.54
SER113	ARG742	2.76	LYS1022	THR506	2.82
PRO246	HIS682	2.52	VAL976	HIS292	2.84 (pi-donor)
GLY213	HIS682	2.49			
PHE212	HIS705	2.61			
ASP112	ARG742	2.45			
ASN220	THR116 0	2.30			
THR20	LYS1171	2.12			
GLU2	LYS1171	2.74			
ASN25	VAL117 6	3.09			
PHE212	ASN724	2.27 (pi-donor)			
**Hydrophobic interactions**					
LYS215	HIS682	2.90	TYR989	TYR83	5.73 (pi-pi T-shaped)
LYS111	ARG703	5.12	PRO1093	LYS11	4.80 (alkyl)
ILE224	TYR107 9	5.42	PRO979	ARG290	4.55 (alkyl)
ILE182	PHE115 6	5.02	VAL975	HIS292	5.01 (pi-alkyl)
			VAL976	HIS292	4.38 (pi-alkyl)
			LYS1022	HIS505	4.03 (pi-alkyl)
**Electrostatic interactions**					
LYS215	GLU110 9	5.01	GLU1137	ARG6	5.29
LYS215	ASP1139	4.54	GLU1137	ARG6	5.16
GLU114	ARG764	5.01	GLU1096	LYS11	4.45
GLU118	ARG764	5.32	ASP944	LYS182	5.33
ASP282	LYS768	5.21	GLU1116	ARG460	4.92
			ASP1018	ARG482	4.83
			GLU1116	ARG482	5.46
			LYS925	ASP205	5.46
			ARG929	ASP260	5.34
			ARG974	GLU888	3.3
			GLU1116	TRP503	4.79 (pi-anion)
**Salt bridges**					
GLU114	ARG742	2.69	ASP942	LYS182	1.93
GLU253	LYS744	2.00	ASP1018	ARG460	1.87
GLU114	ARG764	3.05	ASP1113	LYS479	1.91
GLU2	LYS1171	2.34	GLU1116	LYS479	1.99
GLU2	LYS1171	2.70	ASP1113	LYS479	2.75
GLU2	LYS1171	2.66	GLU1116	ARG482	1.80

			GLU1116	ARG482	2.80
			ARG943	ASP134	3.28
			ARG974	GLU888	1.65

Supplementary Table S6. Effect of random mutations in F14 on the binding of F14 to TLR1/2.

Mpox antigens	Predicted physicochemical characteristics									
	Formula	Amin o acids	Mol. wt.	pI	-vely charged residues	+vely charged residues	Total atoms	Instabilit y index	Aliphati c index	GRAV Y score
**F14**	$C_{353}H_{560}N_{86}O_{134}S_4$	73	8281.06	3.48	21	2	1137	56.28 (u)	112.19	-0.223
**MPXVgp 154**	$C_{365}H_{617}N_{91}O_{127}S_2$	74	8376.57	4.47	19	12	1202	34.62 (s)	109.19	-0.491
**A47R**	$C_{1225}H_{1912}N_{326}O_{384}S_{10}$	240	27671.2 1	4.99	38	26	3857	38.44 (s)	93.33	-0.263
**Cowpox A-type inclusion protein**	$C_{2667}H_{4082}N_{716}O_{792}S_{18}$	512	59425.1 5	5.64	73	54	8275	45.36 (u)	87.21	-0.503
**B11R**	$C_{1512}H_{2313}N_{387}O_{429}S_{15}$	282	33257.2 3	7.60	36	37	4656	43.76 (u)	83.26	-0.364

Supplementary Table S7. Physicochemical characteristics of selected Mpox antigens as predicted by the ProtPARAM server.

Position of mutated residues in F14	Docking score	Binding free energy (ΔG) (kCal mol^{-1})	Binding affinity (K_D) (M)
GLU34	-1825.2	-11.7	2.4e^{-09}
ASP59	-1625.5	-19.2	8e^{-15}
ASP62	-1353.6	-18.2	4.2e^{-14}
ASP63	-1230.0	-8.8	3.8e^{-07}
GLU66	-1946.2	-11.5	3.5e^{-09}
GLU70	-861.0	-9.0	2.5e^{-07}
ILE73	-1710.3	-12.8	3.8e^{-10}

Supplementary Table S8. Antigenicity and allergenicity of selected Mpox antigens as predicted by VaxiJen 2.0 and AllerTOP 2.0 servers.

Selected Mpox antigens	Predicted Antigenicity	Predicted Allergenicity
F14	0.2815	Probable non-allergen
MPXVgp154	1.0183	Probable non-allergen
A47R	0.4608	Probable allergen
Cowpox A-type inclusion protein	0.4762	Probable non-allergen
B11R	0.3983	Probable non-allergen

Supplementary Table S9. Active sites found on F14 protein.

Name of active sites found on F14	Number of active sites found	Site Sequences
N-glycosylation site	1	NFSD
Casein kinase II phosphorylation site	3	STMD, TDIE, SLIE
N-myristoylation site	2	GLSISN, GQQSTM

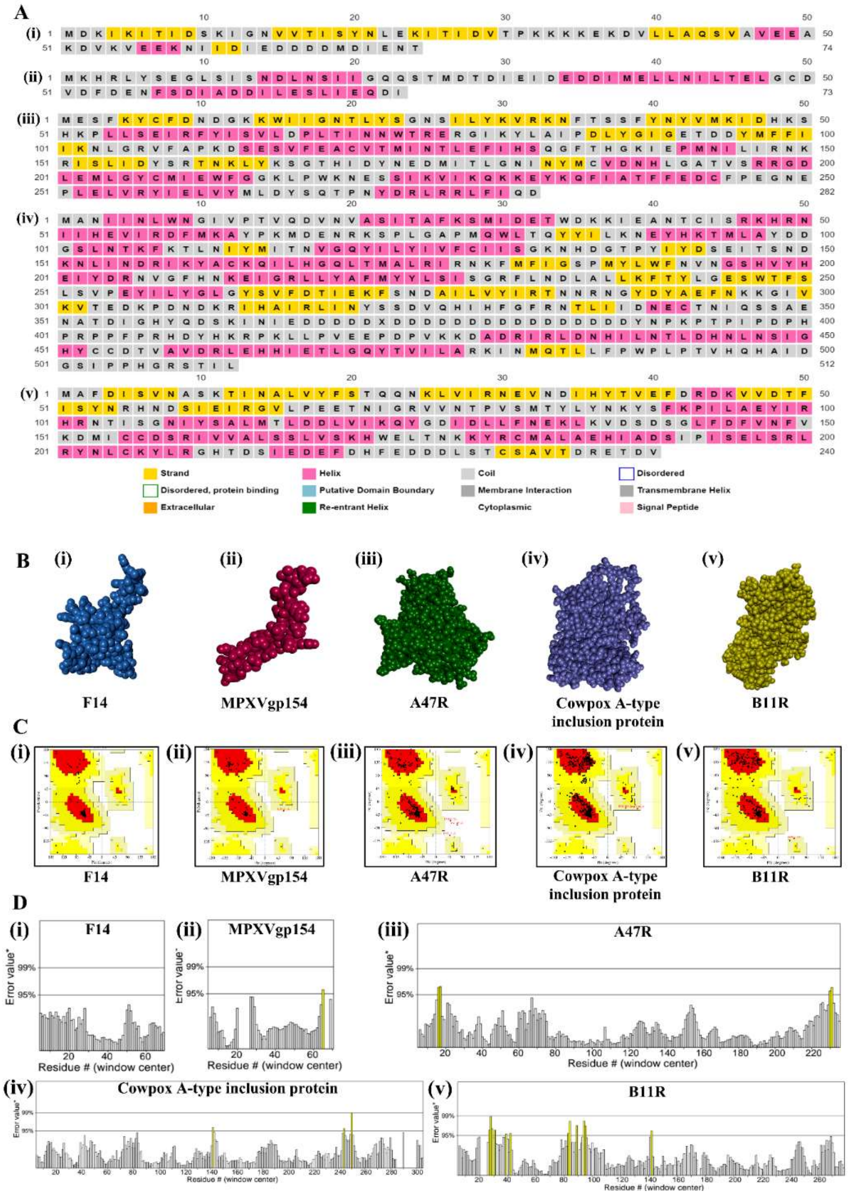

Supplementary Fig. S1. Sequences, structures and stereochemical quality of the 5 selected Mpox antigens. **A**, Amino acid sequences of (i) F14, (ii) MPXVgp154, (iii) A47R, (iv) cowpox A-type inclusion protein and (v) B11R, visualized using MEGA X software. **B**, 3-dimensional space-fill models the 5 MAgs namely (i) F14, (ii) MPXVgp154, (iii) A47R, (iv) cowpox A-type inclusion protein and (v) B11R. **C**, Ramachandran plots of (i) F14, (ii) MPXVgp154, (iii) A47R, (iv) cowpox-A-type inclusion protein and (v) B11R. The respective complexes exhibited 91.2%, 88.6%, 90.7%, 90.2% and 90.9% of residues in the most favored regions of their Ramachandran plots, with a negligible percentage of residues in additionally allowed regions and disallowed regions. **D**, ERRAT plots of (i) F14, (ii) MPXVgp154, (iii) A47R, (iv) cowpox A-type inclusion protein and (v) B11R, were determined to be 100.00%, 98.182%, 98.253%, 91.540% and 95.253% respectively.

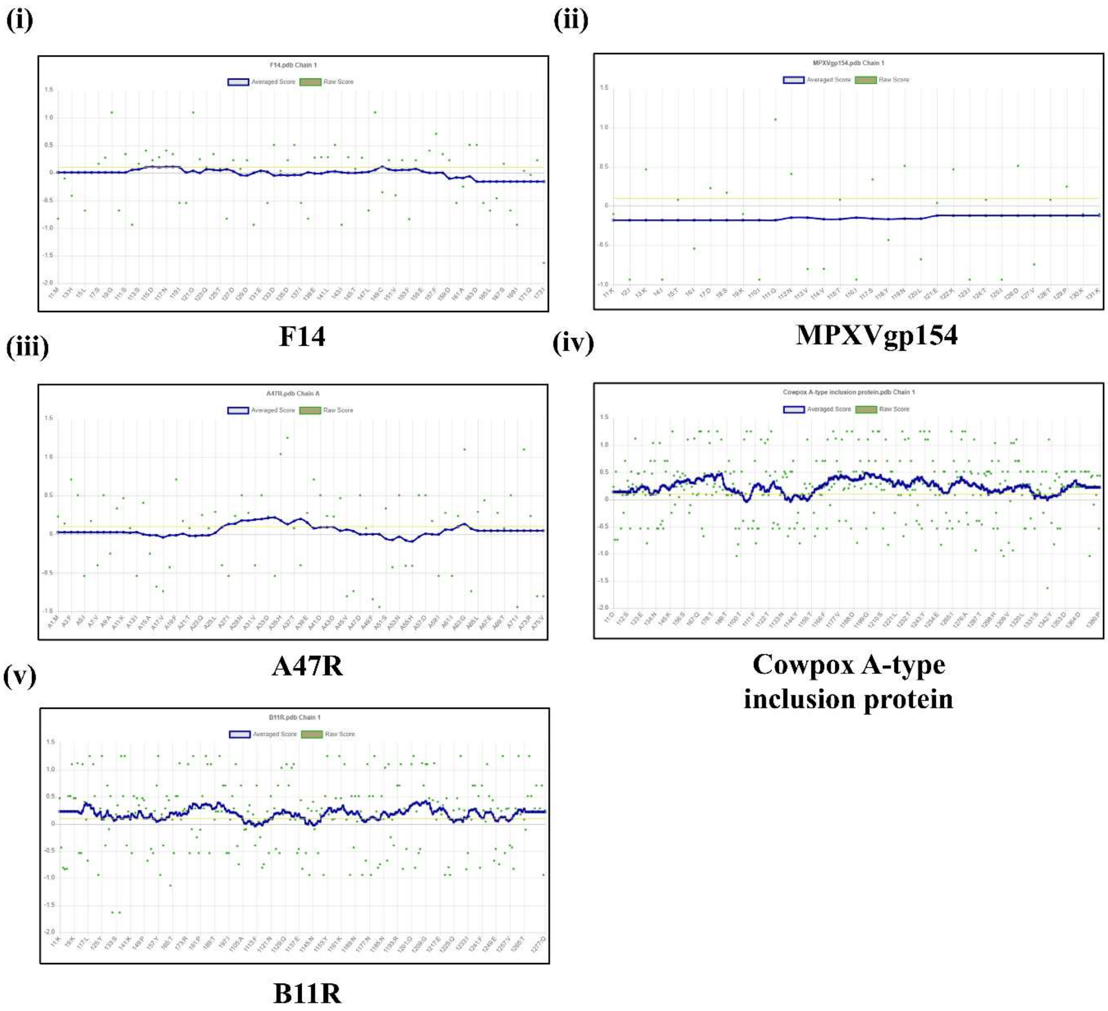

Supplementary Fig. S2. Stereochemical properties of the 5 selected Mpox antigens (i) F14, (ii) MPXVgp154, (iii) A47R, (iv) cowpox A-type inclusion protein and (v) B11R antigens verified through VERIFY3D.

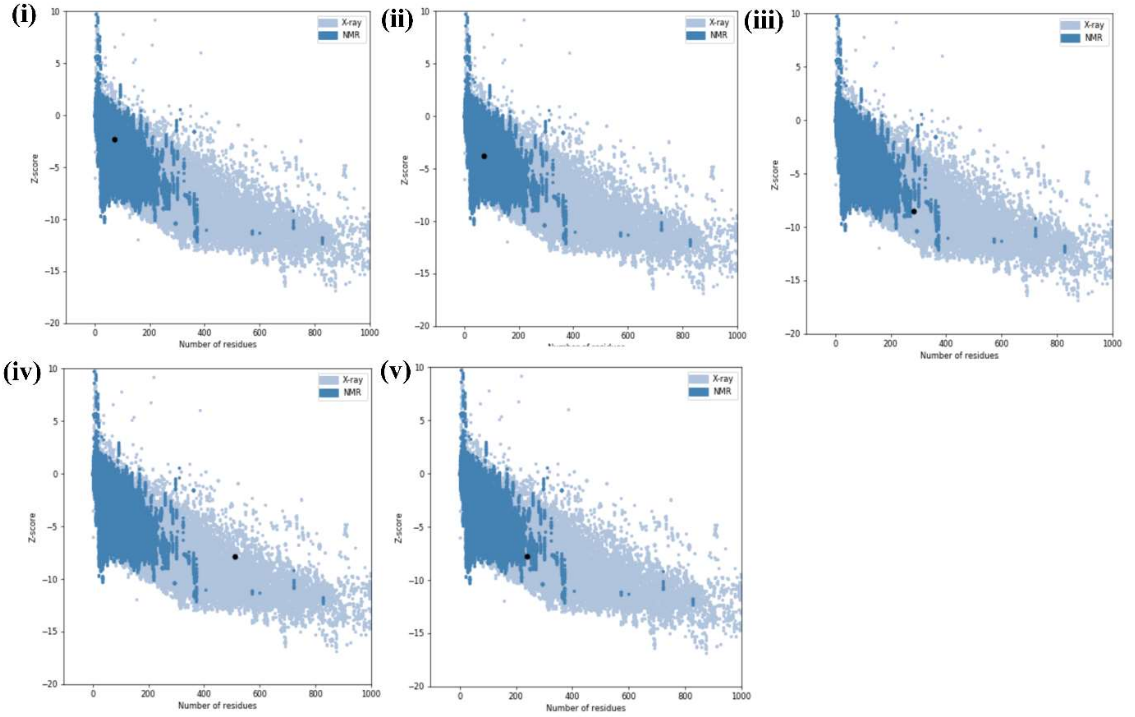

Supplementary Fig. S3. Z-score showing structural stability of (i) F14, (ii) MPXVgp154, (iii) A47R, (iv) cowpox A-type inclusion protein and (v) B11R antigens acquired from the in-silico X-Ray and NMR analyses by ProSA server.

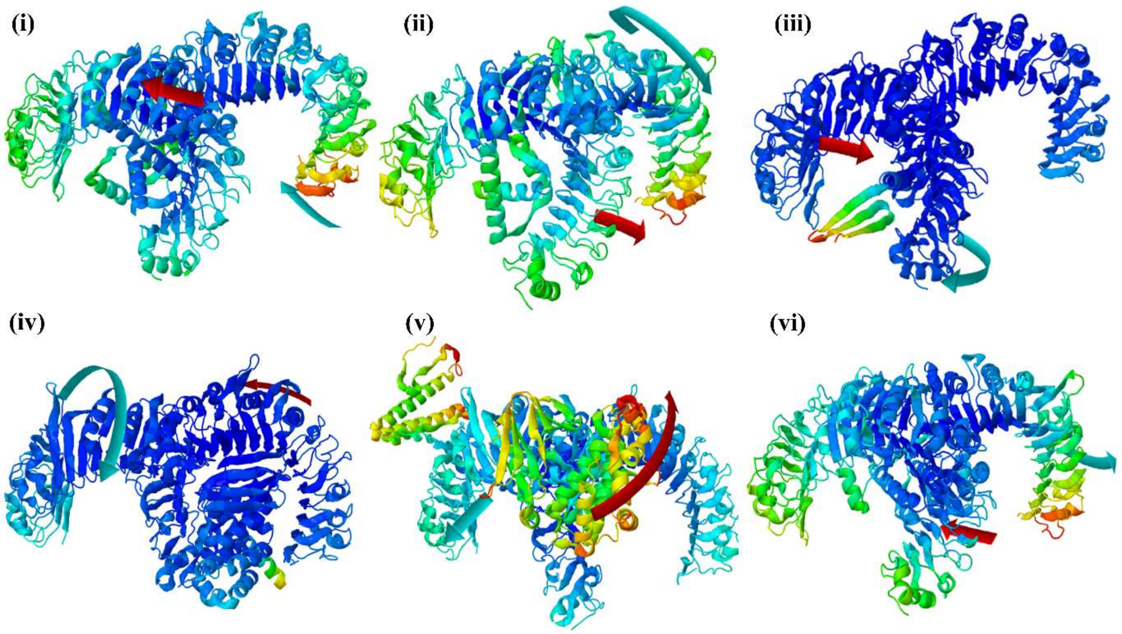

Supplementary Fig. S4. Affine arrow models showing direction of molecular motion of six selected MAg-TLR complexes namely (i) F14 + TLR1/2, (ii) F14 + TLR2/6, (iii) MPXVgp154 + TLR1/2 (iv) A47R + TLR2/6, (v) cowpox A-type inclusion protein + TLR2/6 and (vi) B11R + TLR2/6, as viewed in iMODs server.

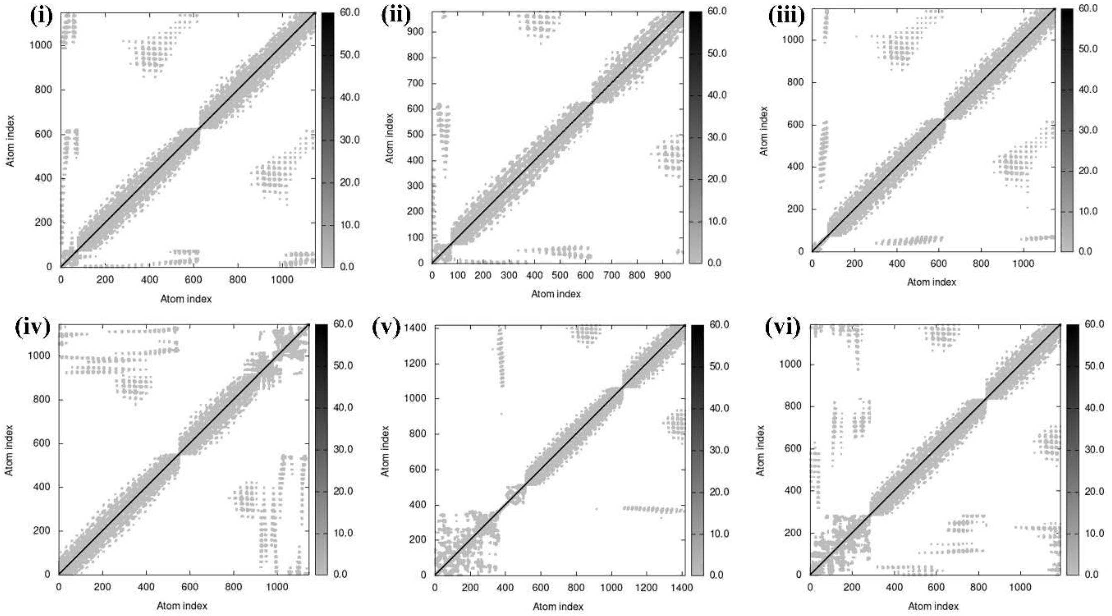

Supplementary Fig. S5. Elastic network maps of (i) F14 + TLR1/2, (ii) F14 + TLR2/6, (iii) MPXVgp154 + TLR1/2 (iv) A47R + TLR2/6, (v) cowpox A-type inclusion protein + TLR2/6 and (vi) B11R + TLR2/6 complexes showing different types of molecular motions conferring stability to the protein complexes.

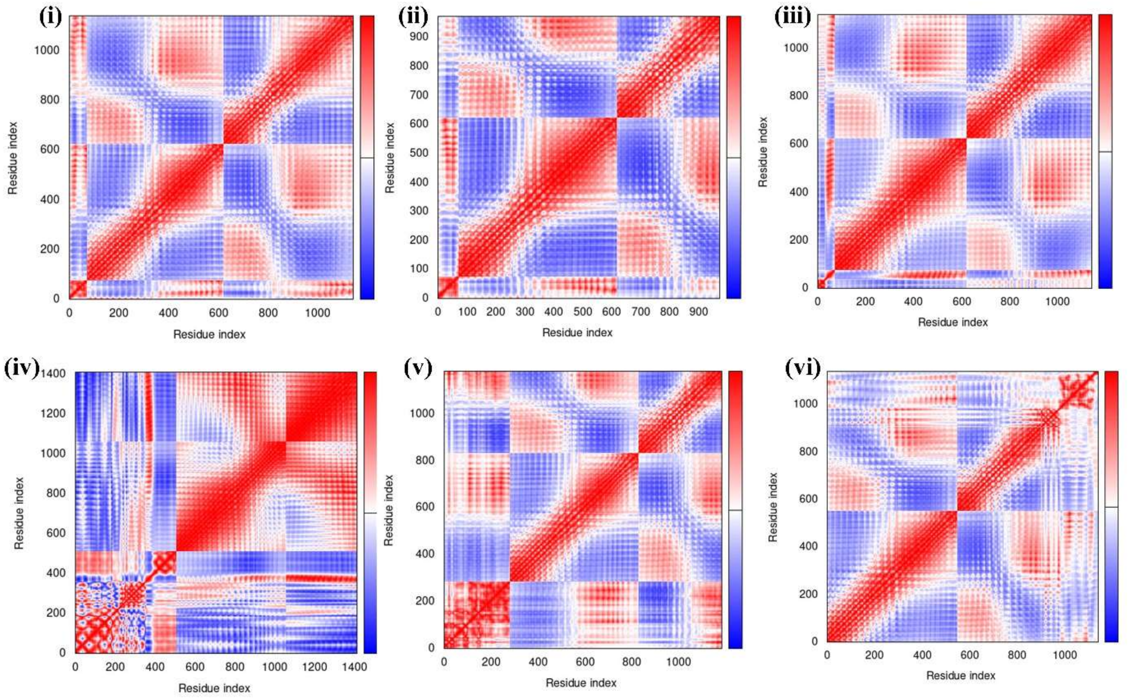

Supplementary Fig. S6. Covariance matrices of (i) F14 + TLR1/2, (ii) F14 + TLR2/6, (iii) MPXVgp154 + TLR1/2 (iv) A47R + TLR2/6, (v) cowpox A-type inclusion protein + TLR2/6 and (vi) B11R + TLR2/6 complexes showing molecular stability.

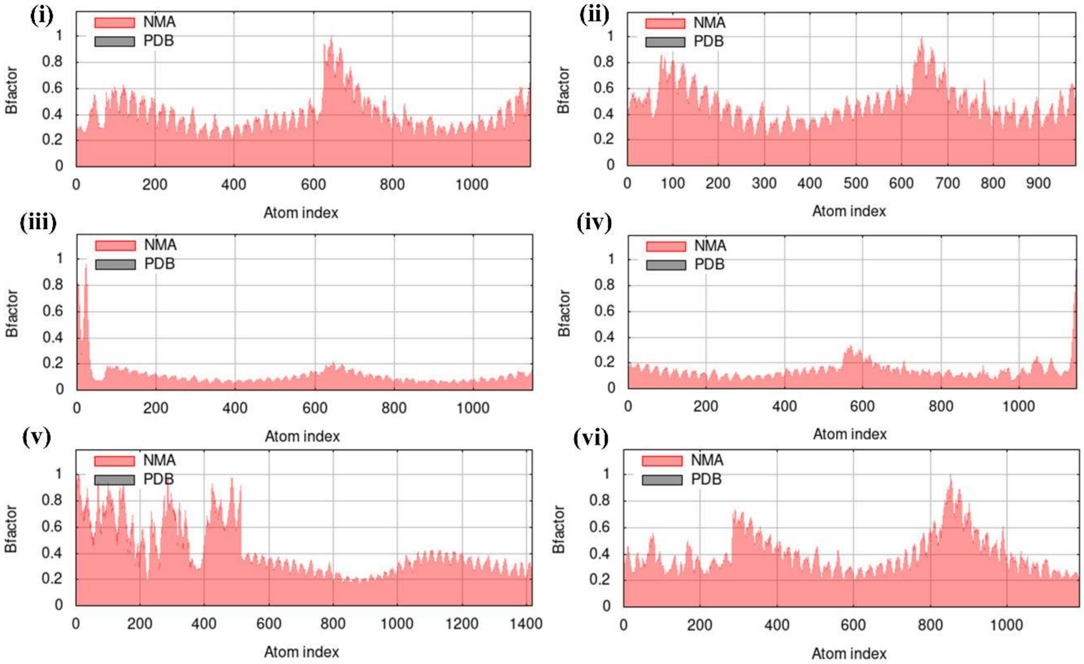

Supplementary Fig. S7. B-factor plots depicting deformation in (i) F14 + TLR1/2, (ii) F14 + TLR2/6, (iii) MPXVgp154 + TLR1/2 (iv) A47R + TLR2/6, (v) cowpox A-type inclusion protein + TLR2/6 and (vi) B11R + TLR2/6 complexes.

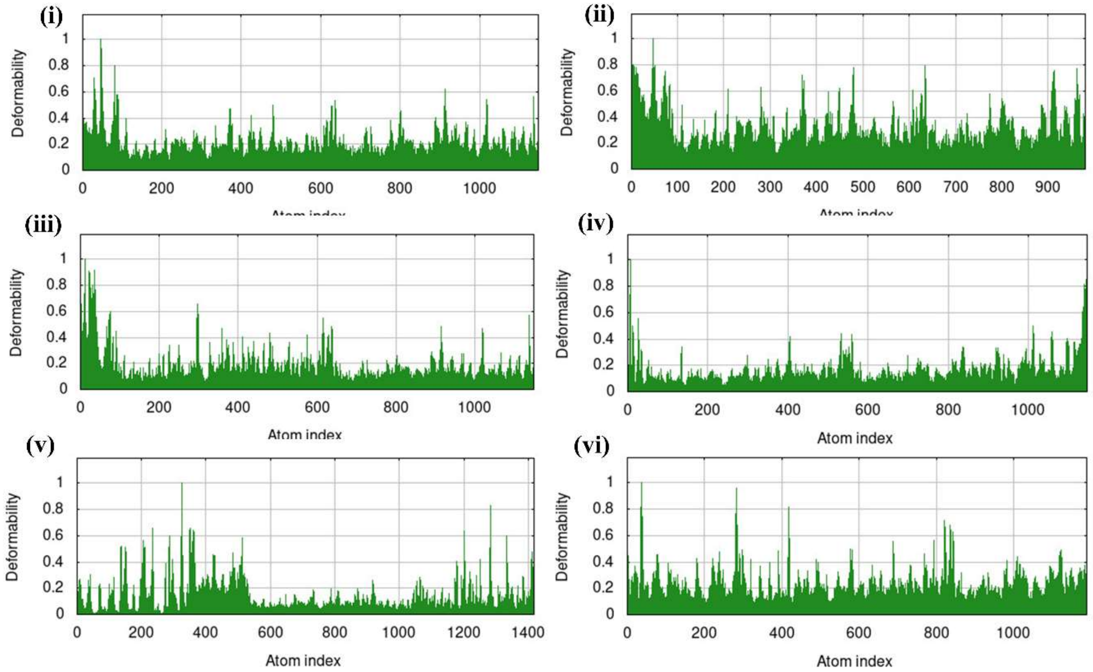

Supplementary Fig. S8. Deformability plots of (i) F14 + TLR1/2, (ii) F14 + TLR2/6, (iii) MPXVgp154 + TLR1/2 (iv) A47R + TLR2/6, (v) cowpox A-type inclusion protein + TLR2/6 and (vi) B11R + TLR2/6 complexes.

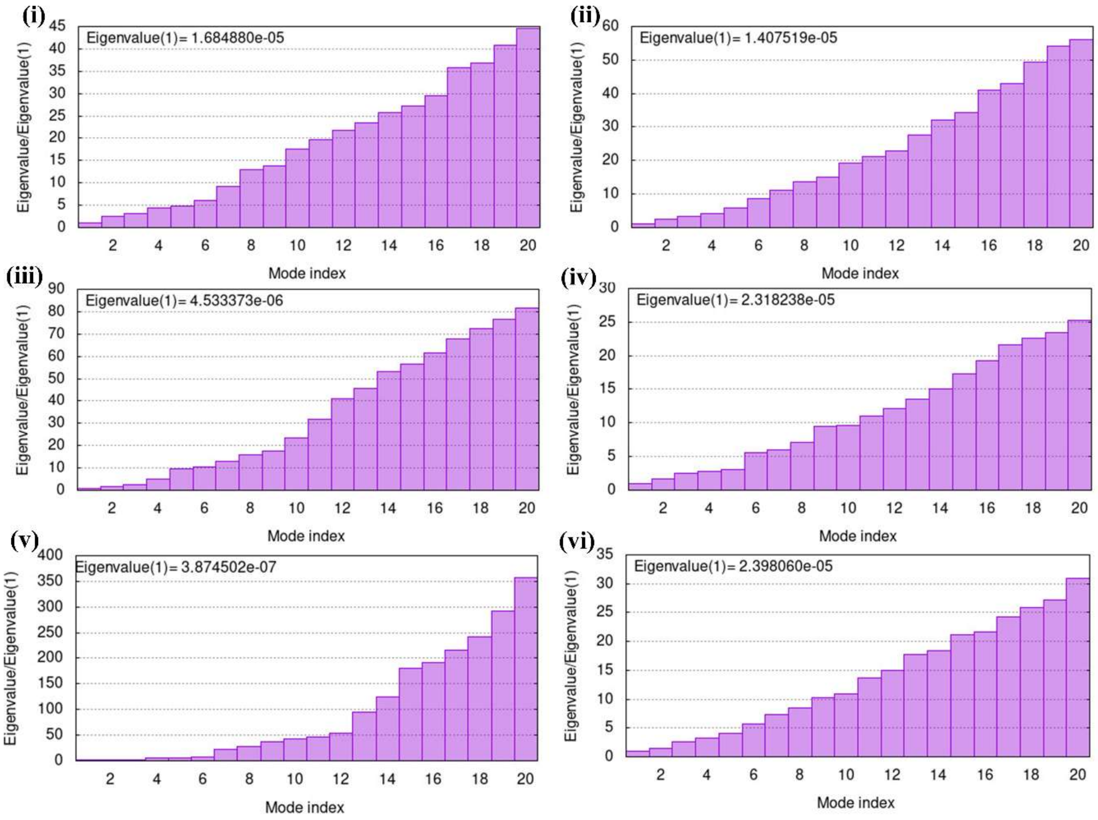

Supplementary Fig. S9. Eigenvalue plots showing comparative stability of (i) F14 + TLR1/2, (ii) F14 + TLR2/6, (iii) MPXVgp154 + TLR1/2 (iv) A47R + TLR2/6, (v) cowpox A-type inclusion protein + TLR2/6 and (vi) B11R + TLR2/6 complexes.

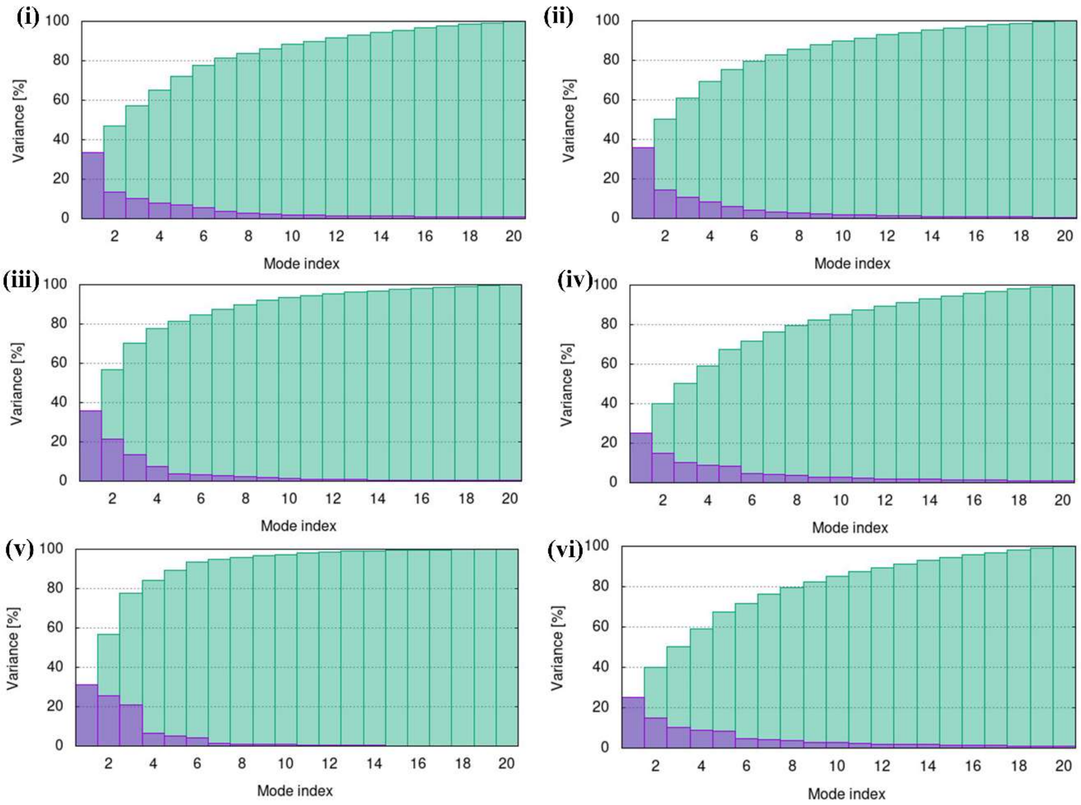

Supplementary Fig. S10. Variance graphs of (i) F14 + TLR1/2, (ii) F14 + TLR2/6, (iii) MPXVgp154 + TLR1/2 (iv) A47R + TLR2/6, (v) cowpox A-type inclusion protein + TLR2/6 and (vi) B11R + TLR2/6 complexes.

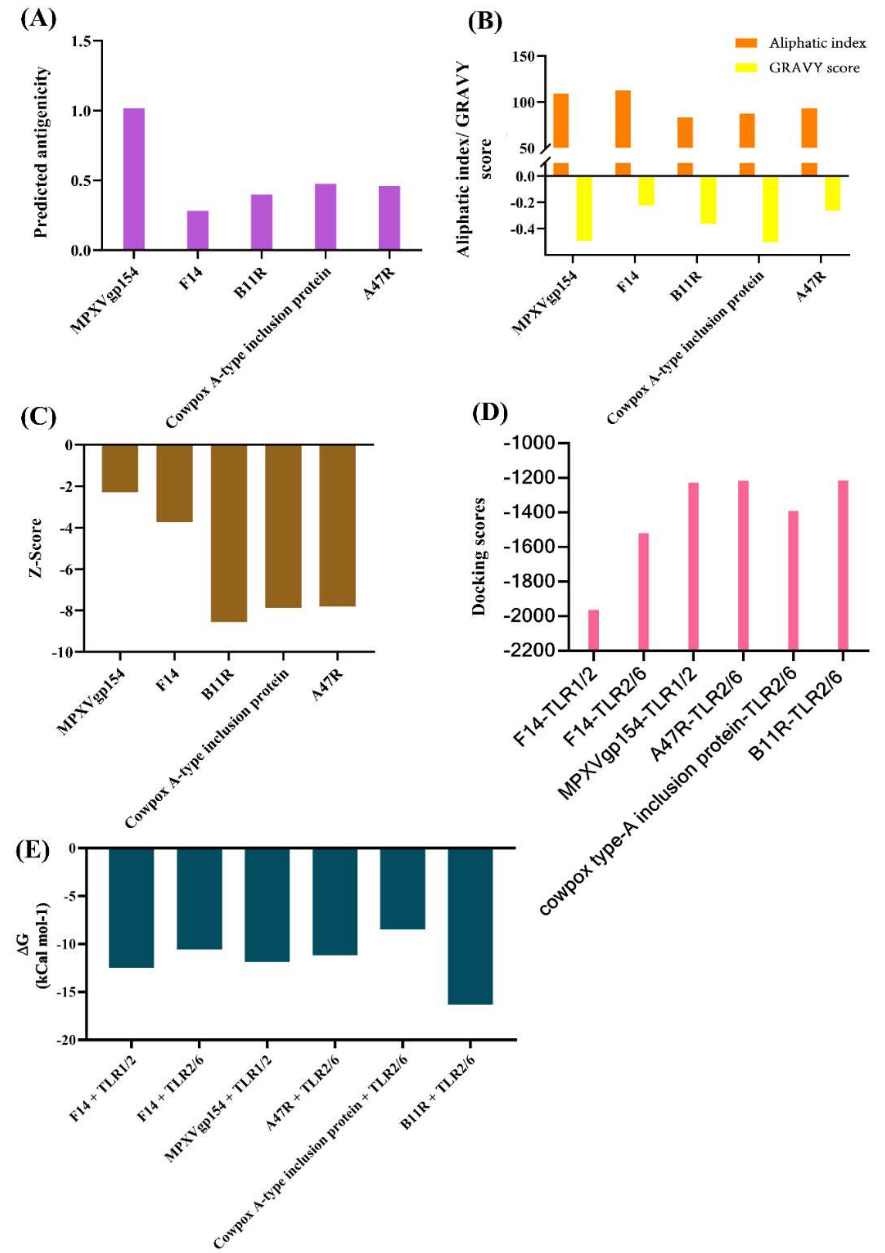

Supplementary Fig. S11. Comparative graphical representations showing (**A**) predicted antigenicity of the 5 Mpox antigens, (**B**) hydrophobicity of 5 Mpox antigens, (**C**) Z-scores of 5 MAgs, (**D**) docking scores of 6 MAg-TLR complexes, (**E**) binding free energy of 6 MAg-TLR complexes.

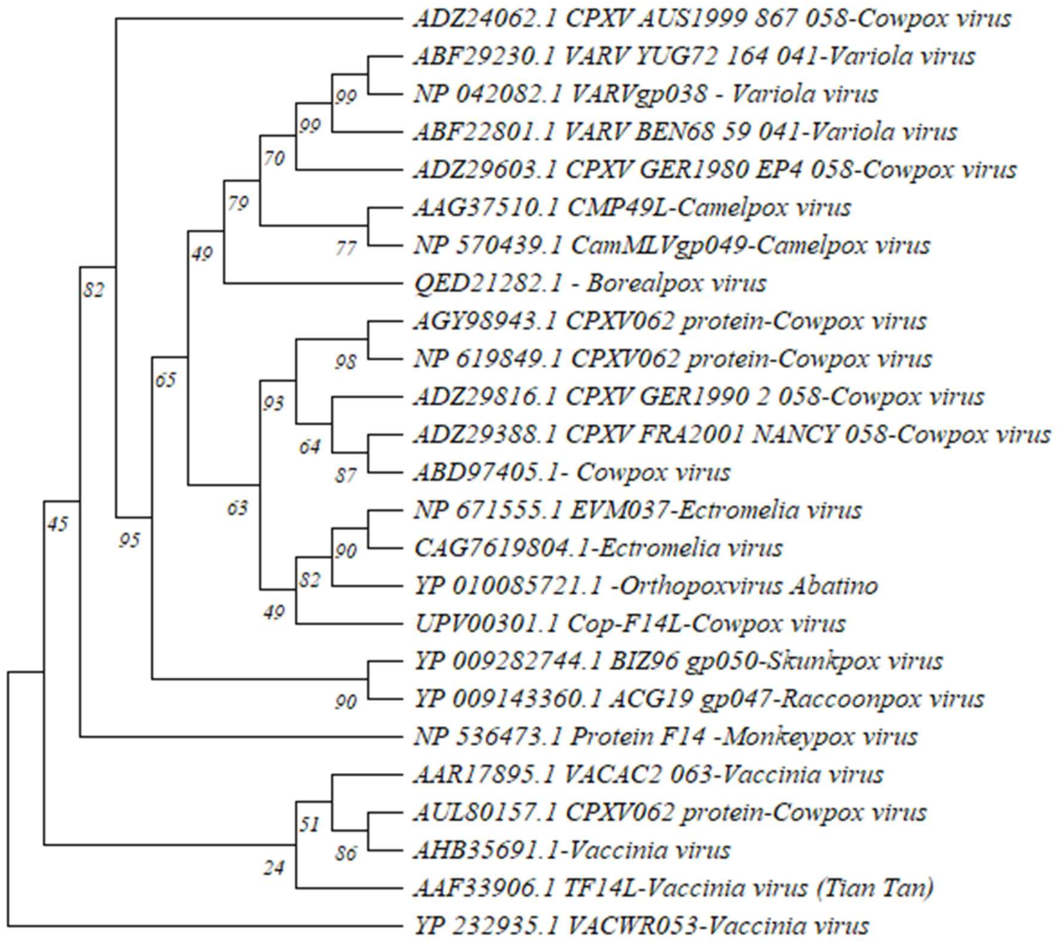

Supplementary Fig. S12. Phylogenetic tree of the Orthopoxviral F14 proteins. The evolutionary history was inferred using the Maximum Parsimony method. Tree #1 out of 4 most parsimonious trees (length = 100) is shown. The consistency index is 0.830000 (0.792683), the retention index is 0.880282 (0.880282), and the composite index is 0.730634 (0.697784) for all sites and parsimony-informative sites (in parentheses). The percentage of replicate trees in which the associated taxa clustered together in the bootstrap test (100 replicates) are shown next to the branches. The MP tree was obtained using the Subtree-Pruning-Regrafting (SPR) algorithm with search level 1 in which the initial trees were obtained by the random addition of sequences (10 replicates). This analysis involved 25 amino acid sequences. There were a total of 85 positions in the final dataset. Evolutionary analyses were conducted in MEGA11.
